# Brain-computer interface paradigms and neural coding

**DOI:** 10.3389/fnins.2023.1345961

**Published:** 2024-01-15

**Authors:** Pengrui Tai, Peng Ding, Fan Wang, Anmin Gong, Tianwen Li, Lei Zhao, Lei Su, Yunfa Fu

**Affiliations:** ^1^Faculty of Information Engineering and Automation, Kunming University of Science and Technology, Kunming, China; ^2^Brain Cognition and Brain-Computer Intelligence Integration Group, Kunming University of Science and Technology, Kunming, China; ^3^School of Information Engineering, Chinese People’s Armed Police Force Engineering University, Xi’an, China; ^4^Faculty of Science, Kunming University of Science and Technology, Kunming, China

**Keywords:** brain-computer interface (BCI) paradigm, neural coding, brain imaging technology, neural decoding, BCI

## Abstract

Brain signal patterns generated in the central nervous system of brain-computer interface (BCI) users are closely related to BCI paradigms and neural coding. In BCI systems, BCI paradigms and neural coding are critical elements for BCI research. However, so far there have been few references that clearly and systematically elaborated on the definition and design principles of the BCI paradigm as well as the definition and modeling principles of BCI neural coding. Therefore, these contents are expounded and the existing main BCI paradigms and neural coding are introduced in the review. Finally, the challenges and future research directions of BCI paradigm and neural coding were discussed, including user-centered design and evaluation for BCI paradigms and neural coding, revolutionizing the traditional BCI paradigms, breaking through the existing techniques for collecting brain signals and combining BCI technology with advanced AI technology to improve brain signal decoding performance. It is expected that the review will inspire innovative research and development of the BCI paradigm and neural coding.

## 1 Introduction

Brain-computer interface (BCI) is a revolutionizing human-computer interaction (Graimann et al., 2013), which directly bypasses peripheral nerves and muscles to establish a new communication and control channel between the brain and external devices (Wolpaw and Wolpaw, 2012). It has the potential to monitor, replace, improve/recover, enhance, and supplement damaged or impaired inputs or outputs of the central nervous system (CNS) (Ramsey and Millán José del, 2020).

In the BCI system, brain signal patterns generated in the CNS of the BCI user are closely related to BCI paradigms and neural coding (Allison et al., 2012), which are the foundation for decoding correctly the user’s intent. Therefore, BCI paradigms and neural coding are critical in BCI research. To date, there have been many references on brain signal processing and classification algorithms in BCI systems. For instance, Lotte et al. (2007, 2018) provided a comprehensive overview of the modern classification algorithms used in electroencephalogram (EEG) -based BCIs, and Bashashati et al. (2007) provided a comprehensive review of the signal processing techniques in BCI systems. However, there have been few references focusing on the definition and design principles of the BCI paradigm as well as the definition and modeling principles of BCI neural coding. For example, Abiri et al. (2019) reviewed EEG-based BCI paradigms, and Xu et al. (2021) reviewed the EEG-based BCI brain coding and decoding mechanisms. In addition to EEG-based BCI paradigms and neural coding, there are also other BCI paradigms and neural coding based on brain imaging techniques, such as intracortical local field potentials (LFP) (Hochberg et al., 2012; Willett et al., 2021, 2023), electroencephalogram (ECoG) (Luo et al., 2022; Branco et al., 2023; Metzger et al., 2023), functional near-infrared spectroscopy (fNIRS) (Abdalmalak et al., 2021; Paulmurugan et al., 2021; Eastmond et al., 2022), functional magnetic resonance imaging (fMRI) (Naselaris et al., 2011; Du et al., 2019), magnetoencephalography (MEG) (Xu et al., 2022; Bu et al., 2023), and hybrid brain-computer interface (hBCI) (Choi et al., 2017; Mussi and Adams, 2022). Therefore, we systematically elaborated on the definition and design principles of the BCI paradigm as well as the definition and modeling principles of BCI neural coding and introduced the existing main BCI paradigms and neural coding. Finally, the challenges and future research directions of the BCI paradigm and neural coding were discussed. It is expected that the review will inspire innovative research and development of the BCI paradigm and neural coding.

## 2 Definition and design principles of the BCI paradigm

### 2.1 Definition of BCI paradigm

Brain-computer interface paradigm is a set of specific mental tasks or external stimuli carefully selected/designed by the BCI developer to represent the user’s intentions under specific brain imaging techniques. The purpose of the BCI paradigm is to “write” the user’s intentions into brain signals, which represent or code the user’s intentions. It is expected that the brain imaging technology used can detect the neural coding of the user’s intentions, laying the foundation for subsequent “reading” or decoding of the user’s intentions (Chavarriaga et al., 2017; Wolpaw et al., 2020). It is worth noting that it is difficult for BCI to decode the arbitrary or random mental activity of the user, as well as the arbitrary or random external stimulus received by the user.

**FIGURE 1:**
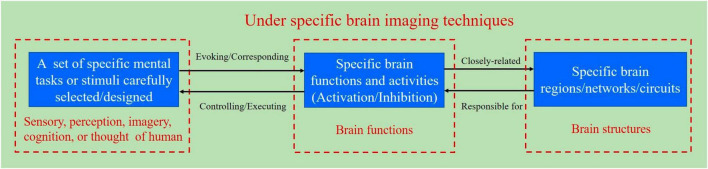
Schematic diagram for the relationship between the BCI paradigm, specific brain functions, and structures.

Specific mental tasks are implicit mental activities, such as motor imagery (MI), visual imagery, speech imagery, mental arithmetic, and reasoning; specific external stimuli are explicit attentional tasks, such as visual, auditory, and tactile stimuli. Specific features of brain signals are induced by mental tasks or external stimuli, and identify specific mental tasks and specific stimuli, which provide the basis for subsequent BCI decoding. Mental tasks or external stimuli correspond to specific brain functions and brain activities, which are closely related to specific brain regions and brain networks/brain circuits. Figure 1 illustrates the relationship between the BCI paradigm, specific brain functions, and structures. Special attention should be paid to the fact that BCI paradigms are usually discussed in the context of specific brain imaging techniques, which means that BCI paradigms are closely related to specific brain imaging techniques.

### 2.2 Principles for designing BCI paradigm

There have been several BCI paradigms so far, such as MI, steady-state visual evoked potential (SSVEP), and P300 paradigms. These paradigms have their advantages and disadvantages, and many researchers are still improving these paradigms. The innovative design of BCI paradigms is one of the critical contents of BCI research. To translate the designed BCI paradigms into practical applications, the principles for designing the BCI paradigm are based on user-centered design (Kübler et al., 2014; Chavarriaga et al., 2017; Lyu et al., 2023) and human factors engineering of BCI (Allison et al., 2012; Kübler et al., 2013, 2020; Liberati et al., 2015; Martin et al., 2018; Kübler, 2020; Ramsey and Millán José del, 2020; Wolpaw et al., 2020; Branco et al., 2021) to evaluate it. The principles for designing the BCI paradigm are proposed in Table 1.

**TABLE 1:** The principles for designing the BCI paradigm.

No.	Design principles
1	CNS signals evoked by BCI paradigm specific tasks should have good separability
2	BCI paradigm tasks are easy for users to perform
3	BCI paradigm tasks are safe for the user
4	BCI paradigm tasks bring the user a good experience and comfort level
5	Tasks specified by BCI paradigm are consistent with tasks controlled by BCI
6	BCI paradigm tasks are designed to the needs of specific user
7	The overall user satisfaction of BCI paradigm tasks is high level

BCI paradigm tasks include specific mental tasks and/or external stimuli.

#### 2.2.1 CNS signals evoked by BCI paradigm specific tasks should have good separability

Brain-computer interface paradigms require users to perform specific mental tasks or receive specific external stimuli. Brain signal features evoked by the user performing the designed BCI paradigm task are significantly differentiable for different mental tasks or external stimuli under specific brain imaging techniques, or the relevant CNS activity better coded the mental tasks or external stimuli designed by the BCI paradigm. Better separability is the basis for subsequently achieving higher BCI decoding accuracy. It is worth noting that specific brain imaging techniques need to be considered in the innovative design of BCI paradigms.

When screening various mental tasks and external stimuli for combination, the classification performance of various mental tasks combinations or external stimuli combinations (Jang et al., 2022) needs to be evaluated to determine the most suitable mental tasks combinations or external stimuli combinations for customized BCI.

#### 2.2.2 BCI paradigm tasks are easy for users to perform

Some mental tasks are easy to perform, while others are not, usually choosing tasks that users are proficient in and natural in their daily life and work. Mental tasks are designed to be as simple as possible, suitable for users, approved, and even enjoyed by users. Easy to perform BCI paradigm tasks can increase user acceptance of BCI technology and promote its translation into practical applications.

#### 2.2.3 BCI paradigm tasks are safe for the user

The brain imaging technology involved in the BCI paradigm is required to be safe for users and not harmful to their physical and mental health (Liberati et al., 2015). In addition, external stimuli have a lower risk of causing brain diseases in users, and mental tasks and external stimuli are less likely to cause excessive fatigue in users, to reduce mental load.

#### 2.2.4 BCI paradigm tasks bring the user a good experience and comfort level

The user experience and comfort of the BCI paradigm are related to the comfort of the sensors used to collect brain signals, as well as the experience and comfort of mental tasks or external stimuli. They are also related to the decoding performance (stability, accuracy, and speed of decoding) under the BCI paradigm, which affects the user’s acceptance of BCI. The BCI paradigm is required to have a high user rating for experience and comfort, which can be evaluated by an experience and comfort questionnaire. At present, the user experience and comfort of the existing BCI paradigm are not high, and the acceptance of BCI by users is not high.

#### 2.2.5 Tasks specified by the BCI paradigm are consistent with tasks controlled by BCI

Brain-computer interface paradigm tasks are designed to avoid non-transparent mappings, which are inconsistencies between mental tasks and control commands, such as the use of left-handed MI to imagine the corresponding commands to control the robot’s movement to the right. Non-transparent mappings may lead to changes in autonomous intentions, which may affect the user’s performance during brain-computer interaction. For this reason, when designing a BCI paradigm task, it is important to consistent the mental task with the task controlled by the BCI.

#### 2.2.6 BCI paradigm tasks are designed to the needs of specific user

During the screening of BCI paradigm tasks, the combination of mental tasks or external stimuli should be designed according to the specific needs of the application, the more mental tasks or external stimuli are not the better. It is necessary to just simply fulfill its requirements. In the case of rehabilitation training for movement disorders, it is appropriate to choose the MI of the corresponding limbs as the paradigm, but if it is to realize the simple communication of “YES” or “NO,” it is not appropriate to use the left or right limb MI as the mental task.

#### 2.2.7 The overall user satisfaction of BCI paradigm tasks is high level

User satisfaction with BCI paradigm tasks is related to several factors that include the above-mentioned BCI paradigms should have good separability, be easy for the user to perform, be safe for the user, bring the user a good experience and comfort level, consistent with tasks controlled by BCI, and fit in the application needs of a specific user, which need to be considered and evaluated comprehensively to design a user-friendly BCI paradigm task.

The BCI paradigm task has a significant impact on whether potential BCI users accept and enjoy using the BCI system, and for this reason, the mental tasks that drive BCI need to be designed and optimized according to the user’s ability characteristics.

## 3 Definition and mechanisms models of BCI neural coding

Based on the definition and design principles of the BCI paradigm elaborated above, the following sections elaborate on the definition and design principles of BCI neural coding, the relationship between BCI paradigm, BCI neural coding, and BCI neural decoding, and the relationship between BCI neural coding, brain neural coding, and computer information coding.

### 3.1 Definition of BCI neural coding

**FIGURE 2:**
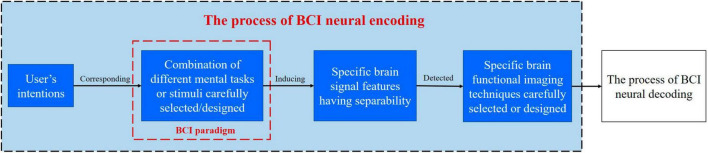
Schematic diagram for the process of BCI neural coding.

Brain-computer interface neural coding refers to the coding of different user intentions into central neural signals under a specific BCI paradigm, which is characterized by distinguishable brain signal features. Brain signals with encoded intentions can be detected by specific brain imaging techniques, at last, user intentions can be recognized by BCI neural decoding algorithms. The process of BCI neural coding is shown in Figure 2.

It is worth noting that BCI neural coding is closely related to the chosen level and parameter settings of brain imaging technology, such that the level of technology used to record the ECoG signals and the hardware settings (including the sampling frequency and hardware filters) affect the types of features that can be extracted and analyses that can be performed (Hill et al., 2012; Shirhatti et al., 2016).

### 3.2 Principles for modeling BCI neural coding

Modeling BCI neural coding requires consideration of specific BCI paradigms, mechanisms of brain neural coding, neural signal features collected by different brain imaging techniques, and efficient decoding of the user’s intentions.

(1) Modeling neural coding under a specific BCI paradigm. Different BCI paradigms, such as SSVEP-BCI, P300-BCI, MI-BCI, and other paradigms have different neural coding models.
(2) Modeling BCI neural coding based on the mechanisms of brain neural coding. The mechanisms of brain neural coding characterize the hypothesized relationships between external stimuli or mental tasks and the response of specific neuronal populations, as well as the relationships between the electrical activity of neurons within neuronal populations (Johnson, 2000; Brown et al., 2004). An implantable BCI neural coding model can be modeled based on these relationships within the mechanisms of brain neural coding.
(3) Considering the features from the time domain, frequency domain, and spatial domain of neural signals collected using different brain imaging techniques when modeling BCI neural coding. Given the different temporal and spatial resolutions of brain imaging techniques, such as EEG, fNIRS, fMRI, MEG, ECoG, intracortical LFP, or Spikes, the measured brain activities (electrical activities of central neurons or metabolic activities of brain tissue) are different.
(4) BCI neural coding model being beneficial for subsequent neural decoding. The purpose of modeling BCI neural coding is to efficiently decode the user’s intentions.

### 3.3 Relationship between BCI paradigm, BCI neural coding, and BCI neural decoding

**FIGURE 3:**
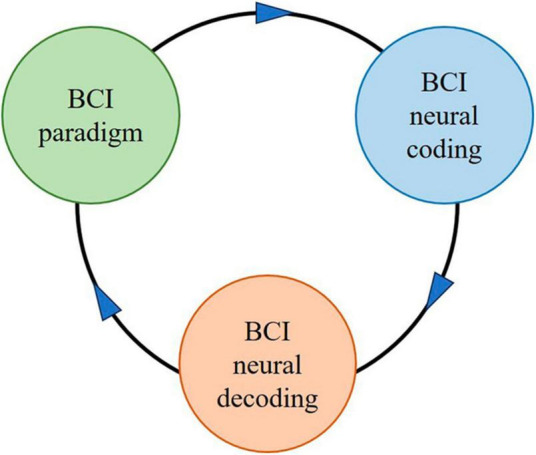
The schematic diagram for the relationship between BCI paradigm, BCI neural coding, and BCI neural decoding.

Brain-computer interface paradigm tasks are usually designed first, then the neural coding under the BCI paradigm is revealed, followed by the extraction of brain signal features from neural coding laws, and finally the neural decoding. The BCI paradigm with BCI neural coding is the basis or premise of BCI decoding. It should be emphasized that in BCI systems, there is no corresponding neural coding without the BCI paradigm, no neural decoding without BCI neural coding, or no high-performance neural decoding performance without good BCI paradigms and neural coding. Figure 3 illustrates the relationship between BCI paradigm, BCI neural coding, and BCI neural decoding.

### 3.4 Relationship between BCI neural coding, brain neural coding, and computer information coding

**FIGURE 4:**
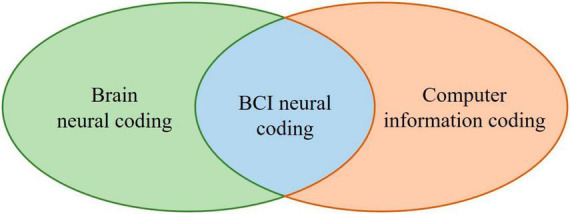
The schematic diagram for the relationship between BCI neural coding, brain neural coding, and computer information coding.

Brain neural coding is the basis of BCI neural coding, which characterizes the hypothesized relationships between external stimuli / mental tasks and the responses of specific neurons or populations of neurons (Johnson, 2000; Brown et al., 2004). According to the theory that sensory and other information is represented in the brain by a network of neurons, it is believed that neurons can encode both digital and analog information (Thorpe, 1990). Computer information coding is the process of converting information from one form or format to another and can be used to represent the relationship of things, which can be represented by numbers, letters, special symbols, or combinations of them, to convert data into codes or coded characters that can be translated into the original data form. Figure 4 illustrates the relationship between BCI neural coding, brain neural coding, and computer information coding.

Inspired by the brain neural coding model in Figure 4, BCI neural coding models can be proposed, as shown in Table 2, and will be reviewed in sections 3.5–3.11. These BCI neural codes will eventually be transformed into computer information coding to be processed by computers.

**TABLE 2:** The BCI neural coding model.

No.	Coding model
1	Frequency/rate coding for BCI
2	Time coding for BCI
3	Phase-of-firing coding for BCI
4	Intracortical neuronal population coding for BCI
5	Correlation coding for BCI
6	Sparse coding for BCI
7	Hybrid coding for BCI

### 3.5 Frequency/rate coding for BCI

Frequency/rate coding for intracortical BCI can be inspired by traditional models of neuronal firing rate coding. In most sensory systems, the firing rate increases, generally non-linearly, with increasing stimulus intensity (Kandel et al., 2000). Since the sequence of action potentials generated by a given stimulus varies from trial to trial, neuronal responses are typically treated statistically or probabilistically. The spike count rate for a single trial can be calculated from Equation (1) (Gerstner and Kistler, 2002).


(1) $$\text{SCR}=\frac{N_{\text{spikes}}}{D_{\text{trial}}}$$

The *D*_trial_ is the duration of a trial, with typical values of 100 ms or 500 ms (Gerstner and Kistler, 2002), and *N*_spikes_ is the number of spikes occurring within the *D*_trial_.

The time-dependent rate of issuance in a time-dependent stimulus can be calculated from Equation (2) (Gerstner and Kistler, 2002).


(2) $${\text{FR}}_{\text{td}}=\frac{N_{K}/K}{△\,t}$$

where *N_K_* is the number spike appearing on all repeated trials from time t and t+Δt, K is the number of retrials, t is the start time relative to the stimulus sequence, and Δt is the time interval, usually in the range of a millisecond or a few milliseconds. The FR_td_applies to both resting and time-dependent stimuli, but it is unlikely to be the coding scheme used by neurons in the brain (Gerstner and Kistler, 2002).

### 3.6 Time coding for BCI

Intracortical BCI time coding can be inspired by time coding models in neural coding (Butts et al., 2007; Gollisch and Meister, 2008). The time resolution of neural coding is on the millisecond time scale, suggesting that precise spike timing is an important element in neural coding (Theunissen and Miller, 1995). For example, the ability of many organisms to discriminate between stimuli (such as visual stimuli, auditory stimuli, tactile stimuli, gustatory stimuli, and olfactory stimuli) on a millisecond time scale suggests that time coding is also a model that functions in sensory systems (Gollisch and Meister, 2008).

The spiking activity features that can be extracted by time coding are time-to-first-spike after the stimulus onset (Victor, 2005; Carleton et al., 2010), phase-of-firing for background oscillations, features based on the second and higher statistical moments of the ISI probability distribution (Kostal et al., 2007), spike randomness, or precisely timed groups of spikes (Thorpe, 1990; Jolivet et al., 2006; Chen et al., 2009).

Information on the time structure of stimulus-evoked spike trains or dispensing rates is determined by the dynamics of the stimulus, the properties of the stimulus, and the nature of the neural coding process (Montemurro et al., 2008). In Equation (2) the code is rate coding if FR_td_changes slowly with time and time coding if it changes rapidly.

### 3.7 Phase-of-firing coding for BCI

Intracortical BCI phase coding can be inspired by phase-of-firing coding models in neural coding (Havenith et al., 2011). Phase-of-firing coding is a neural coding scheme that this type of code takes into account the spike count coding and a time label for each spike according to a time reference based on the phase of local ongoing oscillations at low (Montemurro et al., 2008) or high frequencies (Fries et al., 2007; Havenith et al., 2011). The feature of this code is that neurons adhere to a preferred order of spiking between a group of sensory neurons, resulting in a firing sequence (Havenith et al., 2011). For example, each neuron in the visual cortex has its own preferred/preferred relative firing time during the gamma oscillation cycle.

### 3.8 Intracortical neuronal population coding for BCI

Intracortical BCI neuron population coding can be inspired by the joint activity of multiple neurons to characterize stimuli or mental activity. In BCI intracortical neuronal population coding, each neuron has a distribution of responses over some set of inputs, and the responses of many neurons may be combined to determine corresponding stimulus or mental activity about the inputs. Neuronal population coding captures the essential features of neural coding (Wu et al., 2002).

Experimental studies have revealed that this coding paradigm is widely used in the sensor and motor areas of the brain. for example, Neurons are modulated to the moving/motor direction in the visual area medial time (Maunsell and Van Essen, 1983). However, Individual neurons fire fastest or slower in one direction depending on the distance of the target from the neuron’s “preferred” direction, the vectors of all neurons, and the coding of motion signals.

Typical neuronal population coding involves neurons with Gaussian modulation curves whose mean value varies linearly with stimulus intensity. Positional coding in a population of neurons can be used to encode continuous variables such as joint position, eye position, color, or sound frequency. Rate coding of the entire population ensures higher fidelity and accuracy than rate coding of individual neurons (Mathis et al., 2012).

### 3.9 Correlation coding for BCI

Intracortical BCI correlation coding can be inspired by the correlation coding model of neuronal firing (Panzeri et al., 1999). This model suggests that correlations between action potentials or “spikes” within a spike sequence may carry additional information beyond the simple timing of the spikes (Ahmadi et al., 2021; Zhang and Constandinou, 2023). Correlations may also carry information that is not present in the average firing rate of the two pairs of neurons (Decharms and Merzenich, 1996).

### 3.10 Sparse coding for BCI

Intracortical biometric sparse coding can be inspired by the sparse coding model of neural coding, in which each activity of a stimulus or mental activity is encoded by the strong activation of a relatively small set of neurons, which do not use the full set of available neurons, but rather a subset of them. In the BCI decoding stage, algorithms for sparse signal representation and processing can be used.

The fact that only a few neurons in a population of neurons respond to a given stimulus and that each neuron responds to only a few stimuli out of all possible stimuli, may be a biological selective response. Theoretical studies of sparse distributed memory have shown that sparse coding increases the capacity of associative memory by reducing the overlap between representations (Kanerva, 1988). Experimentally, sparse representations of sensory information have been observed in several systems, including visual (Vision, 2000), auditory (Hromádka et al., 2008), tactile (Crochet et al., 2011), and olfactory (Ito et al., 2008).

Sparsity may be focused on time sparsity, for example, the features extracted from all frequency bands during motion imagery are not all well separable (Olshausen and Field, 1996); It may also focus on spatial sparsity, as in the sparsity of neuronal populations activated, or the sparsity of brain regions/brain networks activated, such that it is the sensory-motor-related brain regions or brain networks that are predominantly activated during MI.

Most sparse coding models are based on linear generative models (Rehn and Sommer, 2007), as shown in Equation (3) (Lee et al., 2006).


(3) $$\vec{\xi }\approx \sum_{j=1}^{n}s_{j}\,\vec{b_{\text{j}}}$$

where $\vec{\xi }\,\epsilon \,{\mathbb{R}}^{k}$ is the set of k-dimensional real input vectors, $\vec{{\text{b}}_{\text{j}}}\,\epsilon \,{\mathbb{R}}^{k}$ is the set of n k-dimensional basis vectors, and $\vec{s}=\left(s_{1},s_{2},\ldots ,s_{j},\ldots ,s_{n}\right)\,\epsilon \,{\mathbb{R}}^{n}$ is the sparse n-dimensional vectors and *s_j_* is the weight of each input vector *b_j_* are combined linearly by Eq. (3) to approximate the inputs. Other models are based on match tracking and dictionary learning (Pati et al., 1993; Davis et al., 1994; Needell and Tropp, 2009).

### 3.11 Hybrid coding for BCI

To better or more fully characterize the relationship between the stimulus / mental activity and the neural response, a combination of several neural coding methods can be considered, which is a hybrid neural coding approach. To more accurately decode the stimulus or mental activity from the neural response, intracortical BCI hybrid coding schemes can combine two or more of the above models (Choi et al., 2017; Mussi and Adams, 2022). For example, global features such as pitch or formant transition profiles can be represented by both rate coding and place coding (Miller and Sachs, 1983).

## 4 Existing main BCI paradigms and neural coding

Brain-computer interface paradigms and neural coding are directly related to specific brain imaging techniques. The existing main BCI paradigms and neural coding models involve brain imaging techniques including the acquisition of intracortical LFP, ECoG, fNIRS, fMRI, MEG, EEG, and hybrid brain imaging techniques, as shown in Table 3.

**TABLE 3:** Existing main BCI paradigms and neural coding.

No.	Existing main BCI paradigms and neural coding
1	Intracortical LFP-BCI paradigms and neural coding
2	ECoG-BCI paradigms and neural coding
3	fMRI-BCI paradigms and neural coding
4	fNIRS-BCI paradigms and neural coding
5	MEG-BCI paradigms and neural coding
6	EEG-BCI paradigms and neural coding
7	Hybrid BCI (hBCI) Paradigms and Coding

### 4.1 Intracortical LFP-BCI paradigms and neural coding

The wrapping of intracortical electrodes has a significant effect on the collection of individual neuronal Spikes but not on LFP (Milekovic et al., 2018, 2019). LFP is expected to be used for long-term cortical control of an artificial device and is a low-frequency signal (<250 Hz) consisting of the sum of all the electrical activity in the region adjacent to the tip of the electrodes implanted in the cortex.

To date, most LFP-based BCI research has been carried out based on typical center-out arm movements (Rickert et al., 2005; Heldman et al., 2006; Wang, 2006), and some studies have employed point-to-point motor tasks (Ahmadi et al., 2021; Zhang and Constandinou, 2023). It has been shown that the M1 tune encodes movement direction (Georgopoulos et al., 1986) and velocity. There is a modest decrease in β-LFP amplitude beforehand movement and a large increase in HF-LFP spectral amplitude during hand movement. The time-domain characterization of LFP can be used to control computer cursors (Kennedy et al., 2004) and to encode motion parameters (Rickert et al., 2005; Ahmadi et al., 2021). Examples of the main existing LFP-BCI paradigms with neural coding studies are shown in Supplementary Table 1.

### 4.2 ECoG-BCI paradigms and neural coding

Electroencephalogram is the recording of the overall activity of a large population of neurons in a localized area by electrodes placed on the supernatural or subdural cortex, with time and spatial resolution of a few milliseconds and millimeters (ECoG is superior to MEG and EEG) (Katzner et al., 2009; Dubey and Ray, 2019), which is less affected by muscle activity and ocular artifacts (Ball et al., 2009), The ECoG has an excellent noise ratio. These advantages favor the coding of stimuli or mental tasks so that potential brain signal features are found to be well discriminated, Therefore, the brain imaging technique of ECoG is better suited for BCI.

The ECoG-BCI frequency coding model is the ECoG power spectrum associated with a specific event (stimulus or mental task), and studies have shown that the selection of appropriate electrodes and power can encode movement trajectories (Jang et al., 2022), and its temporal coding model is the peaks of the raw ECoG signals when time-locked to a specific event (a specific amount of time after stimulus presentation). It has been shown that ECoG high-frequency broadband (200–300 Hz) power variations carry a great deal of information about brain function and are coding information of robustness (Hermes et al., 2011, 2012; Siero et al., 2013, 2014; Vansteensel et al., 2016). In addition, visual, auditory, and tactile evoked ECoG potentials (Brunner et al., 2011; Hermes et al., 2015, 2017; Wittevrongel et al., 2018) and ECoG narrow-band (α, β, and γ) power variations (Crone et al., 1998; Pfurtscheller et al., 2003a; Miller et al., 2007; Kubánek et al., 2009; Brunner et al., 2011; Gunduz et al., 2012; Hermes et al., 2012, 2014; Burke et al., 2013, 2015; Brinkman et al., 2014, 2016) can characterize the function of specific brain regions and brain circuits, Their combination with high-frequency broad-band (Miller et al., 2009, 2016) power changes can sometimes improve decoding performance. Examples of the main existing ECoG-BCI paradigms with neural coding studies are shown in Supplementary Table 2.

### 4.3 fMRI-BCI paradigms and neural coding

Functional magnetic resonance imaging (Ogawa et al., 1990) has high spatial resolution (Logothetis et al., 2001), better robustness and user-friendliness, and individualized flexibility. The ability of fMRI to measure deep brain region structure and activity, map functional connectivity networks, and allow the use of amygdala and ventral striatum with BCI neurofeedback for user (Mehler et al., 2018) has become a core technique for mapping neuroplasticity (Seitz, 2010).

Spatial localization of brain functions using the fMRI-BCI space coding model to produce spatially distinct patterns of brain activation by engaging different combinations of brain regions during the time that subjects are receiving external stimuli or intentionally performing different mental activities (Yoo et al., 2004; Boly et al., 2007; Monti et al., 2010; Senden et al., 2019). Models of fMRI-BCI time coding reliably detect the onset, offset, and duration of single-trial fMRI responses evoked by various mental activities, which is the assignment of specific coding intervals for specific intentions (Monti et al., 2010). Models of fMRI-BCI amplitude coding using different fMRI signal levels within specific brain regions to encode different movement intentions (Yoo et al., 2001). The combination of fMRI-BCI hybrid coding models using the above signal features (spatial, temporal, and amplitude) can increase the degree of freedom to encode different units of information or increase the distinguishability of the evoked brain activation patterns, thus maximizing the decoding accuracy (Sorger et al., 2012). Examples of the main existing fMRI -BCI paradigms with neural coding studies are shown in Supplementary Table 3.

### 4.4 fNIRS-BCI paradigms and neural coding

Functional near-infrared spectroscopy measures the hemodynamic response of brain tissue during resting state (Abdalmalak et al., 2021; Paulmurugan et al., 2021; Eastmond et al., 2022), external stimulus, and mental activity, including changes in oxy-hemoglobin (HbO) and deoxyhemoglobin (HbR) concentration, with the main advantage of good portability, tolerating a certain degree of head movement of the subject, and a favorable ecological effect. fNIRS-BCI can also be used to facilitate the rehabilitation of patients with motor dysfunction and/or cognitive dysfunction, such as those suffering from stroke and spinal cord injury.

The fNIRS-BCI time coding model extracts time-domain features of hemodynamic responses associated with a specific event (such as external stimulus or mental task), for example, the mean, peak, and variance of HbO and HbR (Holper and Wolf, 2011; Hwang et al., 2014; Naseer et al., 2014; Hong et al., 2015). Models of fNIRS -BCI amplitude coding using different fNIRS signal levels within specific brain regions to encode different movement intentions (Coyle et al., 2007; Kaiser et al., 2014). Examples of the main existing fNIRS -BCI paradigms with neural coding studies are shown in Supplementary Table 4.

### 4.5 MEG-BCI paradigms and neural coding

Magnetoencephalography is a non-invasive neuroimaging technique for detecting weak magnetic field changes generated by the electrical activity of central neurons (Xu et al., 2022; Bu et al., 2023). The technique has high time (less than 1 ms) and spatial [2–5 μm (Cetin and Temurtas, 2021)] resolution and low sensitivity to artifacts generated by muscle activity, but its comfort, aesthetics, and ease of use leave much to be desired.

The MEG-BCI time coding model characterizes mental tasks or external stimuli by information such as the peaks of the time-domain waveforms of the MEG signals as well as the time points. The frequency coding model characterizes a specific event (mental activity or external stimulus) by the MEG power spectral features (Mellinger et al., 2007; Chen and Bai, 2009; Halme and Parkkonen, 2016). MEG signals are complex non-linear and non-stationary signals, which the single time or frequency coding model will lose some feature information, and a time-frequency coding model can be used to obtain the relationship between signal frequency over time (Chholak et al., 2019). MEG-BCI space coding models can be used to downsize the data using spatial filtering methods to differentiate between mental activities or external stimuli (Rathee et al., 2021). Examples of the main existing MEG-BCI paradigms with neural coding studies are shown in Supplementary Table 5.

### 4.6 EEG-BCI paradigms and neural coding

#### 4.6.1 MI paradigms and neural coding

Mental tasks involved in MI paradigms include slow, non-fine, non-dexterous MI, fast, fine, and highly dexterous MI, MI involving a unilateral limb, coordinated MI involving multiple limbs, a single or repetitive MI, as well as kinematics or kinetics parameters imagery. The neural coding of MI paradigms can be encoded using (1) time-domain features such as movement-related potential (MRP) or movement-related cortical potential (MRCP) (movement preparation potentials, movement monitoring potentials, and end-of-movement rebound potentials); (2) frequency-domain features coding, such as neural oscillatory power change features of μ/β/γ and other rhythms, which are commonly used event-related-desynchronization/synchronization (ERD/ERS); and (3) space-domain features coding, such as the primary motor area of the hemispheres, the auxiliary motor area, and the premotor area. In addition, the neural coding of some MI paradigms has yet to be studied in depth.

##### 4.6.1.1 Slow, non-fine, non-dexterous MI

Slow, non-fine, and non-dexterous movements usually involve gross limb movements that do not require rapidity, fine coordination, and a high degree of dexterity.

Slow movements are slow and do not require rapid responses or high rates of execution, which include slow walking, strolling, and soothing stretches are all slow movements (Pfurtscheller et al., 2003b; Müller-Putz et al., 2007). Non-fine movements do not require a high degree of fine coordination or precise control of fine muscle groups. Comparatively, they favor holistic and basic movements. Such as simple hand lifting, striding, and simple dance movements (Pfurtscheller et al., 1997; Ramoser et al., 2000; Kaiser et al., 2014). Non-dexterous movements do not require high skills or complex combinations of movements. They focus more on the simplicity and ease of realization of the movements. For example, balancing simple objects, simple stretching, and bending movements (Neuper et al., 2009).

##### 4.6.1.2 Fast, fine, and highly dexterous movement imagery

Fast, fine, and highly dexterous movements usually involve the movement of fine limbs, the execution of which requires fast, accurate movements with a high degree of skill and coordination These types of movements often require long periods of training and practice to achieve a high level of skill.

Rapid movements are executed at a high speed. It requires rapid reaction and movement execution. The users can react quickly in a short period and complete the movement at a high rate. Fine movements require a high degree of precision and care. The users need to control the movement accurately, including the coordination of small muscle groups and precise handling of details. Highly dexterous movements demonstrate exceptional skill and flexibility. The performer can perform the movement with grace and agility. Implantable acquisition of brain signals with high space resolution is usually used to encode and decode such movements, and scalp EEG makes it difficult to encode and decode such MI with stability and high precision.

##### 4.6.1.3 MI involving the unilateral limb

In daily life, some movements involve only the unilateral limb, such as tapping movements of the right or left index finger, internal or external rotation of the wrist, flexion or extension of the wrist, clenching of the fist, pinching of the thumb against the other fingers, and extension of the arm. These movement exercises favor unilateral limb strength, balance, and coordination, and help improve symmetry and motor control (Pfurtscheller et al., 1997; Ramoser et al., 2000; Neuper et al., 2009; Hashimoto and Ushiba, 2013). The difficulty of recognizing different imagined movements in a single limb is greater compared to the recognition of imagined movements in different limbs. For example, recognizing various imagined movements in the affected limb of hemiparetic patients poses a greater challenge.

##### 4.6.1.4 Coordinated MI involving multiple limbs

In daily life, coordinated movement usually involves synergistic movements of multiple limbs to cooperate in time and space to effectively accomplish a desired task (Zhang et al., 2022). Examples include walking and threading a needle. These types of movements usually require some training making good coordination and overall control between limbs (Müller-Putz et al., 2007; Hashimoto and Ushiba, 2013). The neural and decoding of coordinated motor involving at least two limbs is to be studied in depth.

##### 4.6.1.5 A single or repetitive MI

A single MI BCI paradigm is different from the repetitive or continuous MI BCI paradigm, and the BCI coding is different. The Synchronous MI-BCI requires subjects to perform imagery tasks according to a temporal sequence designed by the researcher, and it is typically used to build classification models. The asynchronous MI-BCI is much more challenging, where subjects’ imaginative mental activity can be self-paced rather than performing the imaginative task according to a temporal sequence designed by the researcher.

##### 4.6.1.6 Kinematic or kinetic parameters imagery

The kinematic parameters of the limb include the velocity of movement, trajectory of movement, and time of movement. The kinetic parameters of the limb include the driving force of movement and acceleration of movement. For example, the speed of the right index finger tap (such as slow, medium, and fast), reaching and grasping processes, space navigation, and grip size (Flint et al., 2022). Compared to kinematic parameter imagery, kinetic parameter imagery coding and decoding have been relatively less studied and more difficult.

#### 4.6.2 External stimulus paradigms and neural coding

##### 4.6.2.1 P300-BCI paradigms and neural coding

In the P300-BCI paradigm, the probability of a target/target stimulus (novel stimulus with small probability) is no more than 20%, and the probability of a non-target stimulus (standard stimulus with large probability) is no less than 80%. When a user is exposed to a target stimulus during 220–500 ms (latency) a positive peak of 5–10 microvolts is induced, most significant at the midline location (Pz, Cz, and Fz in the 10/20 international system). This component characterizes the target stimulus. The visual P300-BCI speller was first implemented by Farwell and Donchin (1988), and subsequently, there have been many variants of the P300-BCI paradigm, mainly differences in visual stimulus characterization and presentation. In addition to the visual P300-BCI paradigm, there are also auditory P300-BCI (Furdea et al., 2009; Klobassa et al., 2009) and tactile P300-BCI (Müller-Putz et al., 2006; Brouwer and Van Erp, 2010).

Although the P300-BCI can provide effective input of characters, the practicality still faces challenges. The system’s online transmission rate is low, which makes it difficult to meet the current real-time needs. The inseparability of external stimuli is tied to attention such as vision and hearing, and offline training tends to be prolonged, which causes fatigue to the users.

##### 4.6.2.2 SSVEP-BCI paradigms and neural coding

In the SSVEP-BCI paradigm, when a subject gazes at a visual stimulus of a certain flicker frequency [low band (<12 Hz), middle band (12–30 Hz), and high band (>30 Hz)], a stabilizing potential component of the same frequency as the stimulus frequency or its higher harmonic frequencies is induced. The SSVEP-BCI paradigm can be traced back to as early as a 1995 conference report (McMillan et al., 1995), but is not the paradigm that is now commonly used; the paradigm that is now commonly used comes from a 1999 conference report (Ming and Shangkai, 1999). Subsequently, the SSVEP-BCI paradigm has many innovative designs such as frequency modulation combined with phase and amplitude modulation (Chen et al., 2015; Zhang et al., 2021).

Although SSVEP-BCI has high performance (for example, significant features, stable amplitude, high interference immunity, high information transfer rate, less training, and large instruction set), it requires highly accurate eye control (Herrmann, 2001; Chang et al., 2014), and may lead to subject fatigue when using low blinking frequency (Molina and Mihajlovic, 2010; Müller et al., 2011; Volosyak et al., 2011) the interaction of SSVEP is unnatural, and the user’s satisfaction remains to be further improved. SSVEP-BCIs must essentially have flickering external visual stimuli, and thus cannot be separated from visual attention. Examples of the main existing EEG-BCI paradigms with neural coding studies are shown in Supplementary Table 6.

### 4.7 hBCI paradigms and coding

**FIGURE 5:**
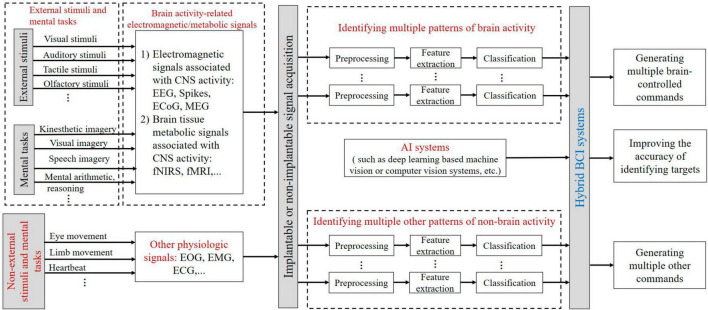
The schematic for hybrid BCI paradigms and coding. EOG, electrooculogram; EMG, electromyogram; ECG, electrocardiogram.

A hybrid Brain-Computer Interface (hBCI) aims to improve the usability or efficacy of BCI systems hBCI consists of a mix of a BCI system (the main system) and an add-on system that assists the BCI (Li et al., 2019). which can be a non-external stimulus or non-psychological task-driven system, or an AI system (e.g., a deep learning-based machine vision or computer vision system) can be mixed to improve the accuracy of target recognition by the main system BCI and increase the number of brain-controlled/other commands. As shown in Figure 5.

As can be seen from Figure 5, BCI paradigms of the main system can be subsets of different external stimuli and mental tasks. For example, the P300 paradigm can be designed by visual, auditory, tactile, or olfactory stimuli according to the Oddball paradigm. The P300 paradigm, the SSVEP paradigm, and the kinesthetic imagery paradigm can be combined. BCI paradigms of the main system induce electromagnetic/metabolic activity signals related to brain activity, and multiple brain activity patterns in these brain signals can encode external stimuli and mental tasks. Non-external stimuli and mental tasks such as eye movement, limb movement, or heartbeat in the additional system can be characterized by other non-brain activity physiological signal patterns. The AI system in the add-on system can enhance the level of intelligence of the main BCI system.

## 5 Challenges and future research directions for BCI paradigms and neural coding

So far, existing BCI paradigms and neural codes have limitations that hinder the translational application of BCI. For this reason, the innovative design and improvement of BCI paradigms and neural codes is one of the key tasks in the development of BCI systems.

### 5.1 User-centered design and evaluation for BCI paradigms and neural coding

The end user of BCI is the user, while the user itself is the source of the CNS signals that drive the BCI. The user is the most complex, active, and highly adaptive subsystem essential to the BCI system. Therefore, BCI systems are the most typical human-in-the-loop systems (the human brain is directly connected or coupled to the machine, a closed-loop system with direct brain-machine interaction), and the design and evaluation (usability and satisfaction) of BCI paradigms and coding need to be user-centered, taking into account BCI human factors engineering (Lyu et al., 2023).

Brain-computer interface paradigms and neural coding are closely related to the neural mechanisms of the user’s mental activities/tasks (Liberati et al., 2015). The performance of a BCI system (such as effectiveness and efficiency) is closely related to the user’s mental activity, such that the performance of a movement-imagery BCI system is largely dependent on the user’s effectiveness or ability to perform MI (Kübler et al., 2014; Martin et al., 2018).

It is worth noting that to evaluate the first principle of BCI paradigm design proposed in section 2.2, which states that CNS signals evoked by BCI paradigm specific tasks should have good separability, any innovatively designed BCI paradigm, and neural coding model typically requires offline data analysis and model establishment, and ultimately must be validated and evaluated by neural decoding in an online BCI system.

### 5.2 Revolutionizing the traditional BCI paradigms

The BCI paradigm, from a perspective of communication principles and technology, is a coding protocol in which brain intentions are encoded into signals generated by neural activity through specific external stimuli or mental tasks.

So far, BCI has been developed for more than 50 years. However, current BCI paradigms are more limited, and the transformation faces great challenges, which need to significantly improve, we need to break through the traditional classical BCI paradigms (such as SSVEP-BCI, P300-BCI, and MI-BCI), and add new BCI paradigms that are more natural and more effective to interact with the user. In recent years, many important advances have been made in the innovation of BCI paradigms (Willett et al., 2021, 2023; Metzger et al., 2023).

#### 5.2.1 Speech-BCI paradigm

Speech is the primary mode of human communication, and the speech BCI paradigm is one of the more natural BCI paradigms. Speech BCI has the potential to decode the neural activity triggered by attempted speech into text or sound, thus promising to restore rapid communication for paralyzed patients (Metzger et al., 2023; Willett et al., 2023).

#### 5.2.2 Handwriting imagery-BCI paradigm for input text

To date, a major focus of BCI research has been the restoration of motor skills to gross limbs such as reaching and grasping or typing with computer cursor clicks. Willett et al. (2021) developed an intracortical brain-computer interface that decodes attempted handwriting actions via neural activity in the hand junction area of the motor cortex and uses a recursive neural network decoding method to translate neural activity in the motor cortex into text in real-time.

### 5.3 Breaking through the existing techniques for collecting brain signals

The performances of the BCI paradigm and neural coding are directly related to the level of brain signal collection technology, which requires a breaking through of brain signal collection technology. How brain signals are acquired is crucial for the BCI paradigm and neural coding, which is related to the quality of the collected signals and the final BCI control effect. With the continuous development of micro-nano processing technology and electrode materials, electrodes for invasive BCI tend to be flexible, miniaturized, high-throughput, and integrated. Currently, the research and development of hydrogel EEG electrodes are more active (Xue et al., 2023), stereotactic EEG (sEEG) (Herff et al., 2020), and in-ear EEG electrodes (Wang et al., 2023) have also made positive progress. In addition, minimally invasive endovascular stent-electrode techniques (Oxley et al., 2016, 2021), minimally invasive local-skull electrophysiological modification methods (Sun et al., 2022), and other schemes have been proposed to innovate brain signal acquisition.

### 5.4 Combining BCI technology with advanced AI technology to improve brain signal decoding performance

**FIGURE 6:**
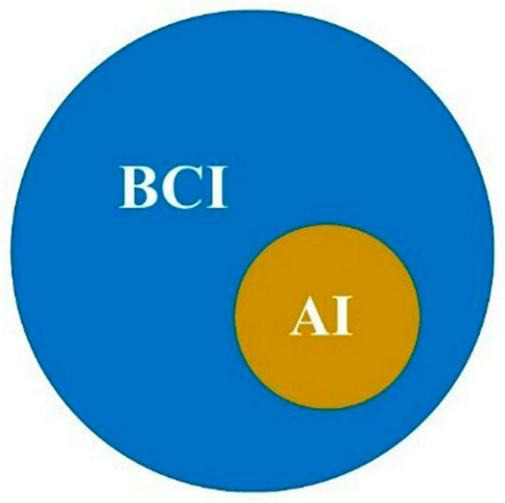
The schematic for combining BCI technology with advanced AI technology.

Currently, classical machine learning still has an advantage in BCI neural decoding, but deep learning also has the potential to enhance BCI decoding performance. Some studies have introduced suitable deep learning algorithms in decoding brain signals, and these studies show that BCI technology combined with advanced AI techniques is expected to significantly improve brain signal decoding performance (Willett et al., 2021, 2023; Metzger et al., 2023). Figure 6 illustrates the introduction of AI into BCI to improve the intelligence of BCI and facilitate the clinical translational application of BCI.

## 6 Conclusion

In the BCI technology system, BCI paradigms and neural coding are some of the key and important contents of BCI research and development. In the paper, the definition of BCI paradigms and seven design principles, as well as the definition and coding model of BCI neural coding, including BCI frequency/rate coding, time coding, phase coding, intracortical neuron population coding, correlation coding, sparse coding, and hybrid coding model are shown more systematically and clearly. The existing main BCI paradigms and neural coding are presented, including intracortical LFP-BCI, ECoG-BCI, fNIRS-BCI, fMRI-BCI, MEG-BCI, EEG-BCI, and hybrid BCI paradigms and neural coding. Finally, user-centered design and evaluation for BCI paradigms and neural coding, revolutionizing the traditional BCI paradigms, breaking through the existing techniques for collecting brain signals and combining BCI technology with advanced AI technology to improve brain signal decoding performance are discussed. It is expected that this paper will inspire the innovative research and development of BCI paradigms and neural coding.

## Author contributions

PT: Writing—original draft. PD: Investigation, Writing—review and editing, Conceptualization, Project administration, Validation. FW: Investigation, Writing—review and editing. AG: Validation, Writing—review and editing. TL: Validation, Writing—review and editing. LZ: Validation, Writing—review and editing. LS: Validation, Writing—review and editing. YF: Conceptualization, Funding acquisition, Investigation, Project administration, Supervision, Validation, Writing—review and editing.

## References

[B1] Abdalmalak A. Milej D. Norton L. Debicki D. B. Owen A. M. Lawrence K. S. (2021). The potential role of fNIRS in evaluating levels of consciousness. *Front. Hum. Neurosci.* 15:703405. 10.3389/fnhum.2021.703405 34305558 PMC8296905

[B2] Abiri R. Borhani S. Sellers E. W. Jiang Y. Zhao X. (2019). A comprehensive review of EEG-based brain–computer interface paradigms. *J. Neural Eng.* 16:011001. 10.1088/1741-2552/aaf12e 30523919

[B3] Ahmadi N. Constandinou T. G. Bouganis C. S. (2021). Impact of referencing scheme on decoding performance of LFP-based brain-machine interface. *J. Neural Eng.* 18:016028. 10.1088/1741-2552/abce3c 33242850

[B4] Allison B. Z. Dunne S. Leeb R. Millan J. Nijholt A. (2012). *Towards practical brain-computer interfaces: Bridging the gap from research to real-world applications.* Berlin: Springer Science & Business Media.

[B5] Ball T. Kern M. Mutschler I. Aertsen A. Schulze-Bonhage A. (2009). Signal quality of simultaneously recorded invasive and non-invasive EEG. *Neuroimage* 46 708–716. 19264143 10.1016/j.neuroimage.2009.02.028

[B6] Bashashati A. Fatourechi M. Ward R. K. Birch G. E. (2007). A survey of signal processing algorithms in brain–computer interfaces based on electrical brain signals. *J. Neural Eng.* 4:R32. 10.1088/1741-2560/4/2/R03 17409474

[B7] Boly M. Coleman M. R. Davis M. H. Hampshire A. Bor D. Moonen G. (2007). When thoughts become action: An fMRI paradigm to study volitional brain activity in non-communicative brain injured patients. *Neuroimage* 36 979–992. 10.1016/j.neuroimage.2007.02.047 17509898

[B8] Branco M. P. Geukes S. H. Aarnoutse E. J. Ramsey N. F. Vansteensel M. J. (2023). Nine decades of electrocorticography: A comparison between epidural and subdural recordings. *Eur. J. Neurosci.* 57 1260–1288. 10.1111/ejn.15941 36843389

[B9] Branco M. P. Pels E. G. Sars R. H. Aarnoutse E. J. Ramsey N. F. Vansteensel M. J. (2021). Brain-computer interfaces for communication: Preferences of individuals with locked-in syndrome. *Neurorehabil. Neural Repair* 35 267–279. 33530868 10.1177/1545968321989331 PMC7934157

[B10] Brinkman L. Stolk A. Dijkerman H. C. Lange F. P. Toni I. (2014). Distinct roles for alpha-and beta-band oscillations during mental simulation of goal-directed actions. *J. Neurosci.* 34 14783–14792. 10.1523/JNEUROSCI.2039-14.2014 25355230 PMC4212072

[B11] Brinkman L. Stolk A. Marshall T. R. Esterer S. Sharp P. Dijkerman H. C. (2016). Independent causal contributions of alpha-and beta-band oscillations during movement selection. *J. Neurosci.* 36 8726–8733. 10.1523/JNEUROSCI.0868-16.2016 27535917 PMC4987441

[B12] Brouwer A. M. Van Erp J. B. (2010). A tactile P300 brain-computer interface. *Front. Neurosci.* 4:1440. 10.3389/fnins.2010.00019 20582261 PMC2871714

[B13] Brown E. N. Kass R. E. Mitra P. P. (2004). Multiple neural spike train data analysis: State-of-the-art and future challenges. *Nat. Neurosci.* 7 456–461. 10.1038/nn1228 15114358

[B14] Brunner P. Ritaccio A. L. Emrich J. F. Bischof H. Schalk G. (2011). Rapid communication with a “P300” matrix speller using electrocorticographic signals (ECoG). *Front. Neurosci.* 5:5. 10.3389/fnins.2011.00005 21369351 PMC3037528

[B15] Bu Y. Harrington D. L. Lee R. R. Shen Q. Angeles-Quinto A. Ji Z. (2023). Magnetoencephalogram-based brain–computer interface for hand-gesture decoding using deep learning. *Cereb. Cortex* 33 8942–8955. 10.1093/cercor/bhad173 37183188

[B16] Burke J. F. Merkow M. B. Jacobs J. Kahana M. J. Zaghloul K. A. (2015). Brain computer interface to enhance episodic memory in human participants. *Front. Hum. Neurosci.* 8:1055. 10.3389/fnhum.2014.01055 25653605 PMC4299435

[B17] Burke J. F. Zaghloul K. A. Jacobs J. Williams R. B. Sperling M. R. Sharan A. D. (2013). Synchronous and asynchronous theta and gamma activity during episodic memory formation. *J. Neurosci.* 33 292–304. 10.1523/JNEUROSCI.2057-12.2013 23283342 PMC3711714

[B18] Butts D. A. Weng C. Jin J. Yeh C. Lesica N. A. Alonso J. (2007). Temporal precision in the neural code and the timescales of natural vision. *Nature* 449 92–95. 17805296 10.1038/nature06105

[B19] Carleton A. Accolla R. Simon S. A. (2010). Coding in the mammalian gustatory system. *Trends Neurosci.* 33 326–334. 20493563 10.1016/j.tins.2010.04.002 PMC2902637

[B20] Cetin O. Temurtas F. (2021). A comparative study on classification of magnetoencephalography signals using probabilistic neural network and multilayer neural network. *Soft Comput.* 25 2267–2275.

[B21] Chang M. H. Baek H. J. Lee S. M. Park K. S. (2014). An amplitude-modulated visual stimulation for reducing eye fatigue in SSVEP-based brain–computer interfaces. *Clin. Neurophysiol.* 125 1380–1391. 10.1016/j.clinph.2013.11.016 24368034

[B22] Chavarriaga R. Fried-Oken M. Kleih S. Lotte F. Scherer R. (2017). Heading for new shores! Overcoming pitfalls in BCI design. *Brain Comput. Interfaces* 4 60–73. 10.1080/2326263X.2016.1263916 29629393 PMC5884128

[B23] Chen G. Wang L. P. Tsien J. Z. (2009). Neural population-level memory traces in the mouse hippocampus. *PLoS One* 4:e8256. 10.1371/journal.pone.0008256 20016843 PMC2788416

[B24] Chen X. Bai O. (2009). “Towards multi-dimensional robotic control via noninvasive brain-computer interface,” in *Proceedings of the 2009 ICME international conference on complex medical engineering*, (Piscataway, NJ), 1–5.

[B25] Chen X. Wang Y. Gao S. Jung T. Gao X. (2015). Filter bank canonical correlation analysis for implementing a high-speed SSVEP-based brain–computer interface. *J. Neural Eng.* 12:046008. 10.1088/1741-2560/12/4/046008 26035476

[B26] Chholak P. Niso G. Maksimenko V. A. Kurkin S. A. Frolov N. S. Pitsik E. N. (2019). Visual and kinesthetic modes affect motor imagery classification in untrained subjects. *Sci. Rep.* 9:9838. 10.1038/s41598-019-46310-9 31285468 PMC6614413

[B27] Choi I. Rhiu I. Lee Y. Yun M. H. Nam C. S. (2017). A systematic review of hybrid brain-computer interfaces: Taxonomy and usability perspectives. *PLoS One* 12:e0176674. 10.1371/journal.pone.0176674 28453547 PMC5409179

[B28] Coyle S. M. Ward T. E. Markham C. M. (2007). Brain–computer interface using a simplified functional near-infrared spectroscopy system. *J. Neural Eng.* 4:219. 10.1088/1741-2560/4/3/007 17873424

[B29] Crochet S. Poulet J. F. Kremer Y. Petersen C. C. (2011). Synaptic mechanisms underlying sparse coding of active touch. *Neuron* 69 1160–1175. 10.1016/j.neuron.2011.02.022 21435560

[B30] Crone N. E. Miglioretti D. L. Gordon B. Lesser R. P. (1998). Functional mapping of human sensorimotor cortex with electrocorticographic spectral analysis. II. Event-related synchronization in the gamma band. *Brain* 121 2301–2315. 10.1093/brain/121.12.2301 9874481

[B31] Davis G. M. Mallat S. G. Zhang Z. (1994). Adaptive time-frequency decompositions. *Opt. Eng.* 33 2183–2191.

[B32] Decharms R. C. Merzenich M. M. (1996). Primary cortical representation of sounds by the coordination of action-potential timing. *Nature* 381 610–613. 8637597 10.1038/381610a0

[B33] Du C. Li J. Huang L. He H. (2019). Brain encoding and decoding in fMRI with bidirectional deep generative models. *Engineering* 5 948–953.

[B34] Dubey A. Ray S. (2019). Cortical electrocorticogram (ECoG) is a local signal. *J. Neurosci.* 39 4299–4311. 30914446 10.1523/JNEUROSCI.2917-18.2019 PMC6538865

[B35] Eastmond C. Subedi A. De S. Intes X. (2022). Deep learning in fNIRS: A review. *Neurophotonics* 9 041411–041411. 35874933 10.1117/1.NPh.9.4.041411 PMC9301871

[B36] Farwell L. A. Donchin E. (1988). Talking off the top of your head: Toward a mental prosthesis utilizing event-related brain potentials. *Electroencephalogr. Clin. Neurophysiol.* 70 510–523. 10.1016/0013-4694(88)90149-6 2461285

[B37] Flint R. D. Li Y. Wang P. Vaidya M. Barry A. Ghassemi M. (2022). Noninvasively recorded high-gamma signals improve synchrony of force feedback in a novel neurorehabilitation brain–machine interface for brain injury. *J. Neural Eng.* 19:036024. 10.1088/1741-2552/ac7004 35576911 PMC9728942

[B38] Fries P. Nikoliæ D. Singer W. (2007). The gamma cycle. *Trends Neurosci.* 30 309–316. 17555828 10.1016/j.tins.2007.05.005

[B39] Furdea A. Halder S. Krusienski D. J. Bross D. Nijboer F. Birbaumer N. (2009). An auditory oddball (P300) spelling system for brain-computer interfaces. *Psychophysiology* 46 617–625. 10.1111/j.1469-8986.2008.00783.x 19170946

[B40] Georgopoulos A. P. Schwartz A. B. Kettner R. E. (1986). Neuronal population coding of movement direction. *Science* 233 1416–1419. 3749885 10.1126/science.3749885

[B41] Gerstner W. Kistler W. M. (2002). *Spiking neuron models: Single neurons, populations, plasticity.* Cambridge: Cambridge University Press.

[B42] Gollisch T. Meister M. (2008). Rapid neural coding in the retina with relative spike latencies. *Science* 319 1108–1111. 10.1126/science.1149639 18292344

[B43] Graimann B. Allison B. Pfurtscheller G. (2013). *Brain-computer interfaces: Revolutionizing human-computer interaction.* Berlin: Springer Publishing Company.

[B44] Gunduz A. Brunner P. Daitch A. Leuthardt E. C. Ritaccio A. L. Pesaran B. (2012). Decoding covert spatial attention using electrocorticographic (ECoG) signals in humans. *Neuroimage* 60 2285–2293. 10.1016/j.neuroimage.2012.02.017 22366333 PMC3321088

[B45] Halme H. L. Parkkonen L. (2016). Comparing features for classification of MEG responses to motor imagery. *PLoS One* 11:e0168766. 10.1371/journal.pone.0168766 27992574 PMC5161474

[B46] Hashimoto Y. Ushiba J. (2013). EEG-based classification of imaginary left and right foot movements using beta rebound. *Clin. Neurophysiol.* 124 2153–2160. 10.1016/j.clinph.2013.05.006 23757379

[B47] Havenith M. N. Yu S. Biederlack J. Chen N. H. Singer W. Nikolić D. (2011). Synchrony makes neurons fire in sequence, and stimulus properties determine who is ahead. *J. Neurosci.* 31 8570–8584. 10.1523/JNEUROSCI.2817-10.2011 21653861 PMC6623348

[B48] Heldman D. A. Wang W. Chan S. S. Moran D. W. (2006). Local field potential spectral tuning in motor cortex during reaching. *IEEE Trans. Neural Syst. Rehabil. Eng.* 14 180–183. 16792288 10.1109/TNSRE.2006.875549

[B49] Herff C. Krusienski D. J. Kubben P. (2020). The potential of stereotactic-EEG for brain-computer interfaces: Current progress and future directions. *Front. Neurosci.* 14:123. 10.3389/fnins.2020.00123 32174810 PMC7056827

[B50] Hermes D. Miller K. J. Vansteensel M. J. Edwards E. Ferrier C. H. Bleichner M. G. (2014). Cortical theta wanes for language. *Neuroimage* 85 738–748. 10.1016/j.neuroimage.2013.07.029 23891904

[B51] Hermes D. Miller K. J. Wandell B. A. Winawer J. (2015). Stimulus dependence of gamma oscillations in human visual cortex. *Cereb. Cortex* 25 2951–2959. 24855114 10.1093/cercor/bhu091 PMC4537439

[B52] Hermes D. Siero J. C. Aarnoutse E. J. Leijten F. S. Petridou N. Ramsey N. F. (2012). Dissociation between neuronal activity in sensorimotor cortex and hand movement revealed as a function of movement rate. *J. Neurosci.* 32 9736–9744. 22787059 10.1523/JNEUROSCI.0357-12.2012 PMC6622254

[B53] Hermes D. Trenité D. G. Winawer J. (2017). Gamma oscillations and photosensitive epilepsy. *Curr. Biol.* 27 R336–R338. 28486114 10.1016/j.cub.2017.03.076 PMC5438467

[B54] Hermes D. Vansteensel M. J. Albers A. M. Bleichner M. G. Benedictus M. R. Orellana C. M. (2011). Functional MRI-based identification of brain areas involved in motor imagery for implantable brain–computer interfaces. *J. Neural Eng.* 8:025007. 10.1088/1741-2560/8/2/025007 21436535

[B55] Herrmann C. S. (2001). Human EEG responses to 1–100 Hz flicker: Resonance phenomena in visual cortex and their potential correlation to cognitive phenomena. *Exp. Brain Res.* 137 346–353. 10.1007/s002210100682 11355381

[B56] Hill N. J. Gupta D. Brunner P. Gunduz A. Adamo M. A. Ritaccio A. (2012). Recording human electrocorticographic (ECoG) signals for neuroscientific research and real-time functional cortical mapping. *J. Vis. Exp.* 26:e3993. 10.3791/3993 22782131 PMC3471287

[B57] Hochberg L. R. Bacher D. Jarosiewicz B. Masse N. Y. Simeral J. D. Vogel J. (2012). Reach and grasp by people with tetraplegia using a neurally controlled robotic arm. *Nature* 485 372–375. 22596161 10.1038/nature11076 PMC3640850

[B58] Holper L. Wolf M. (2011). Single-trial classification of motor imagery differing in task complexity: A functional near-infrared spectroscopy study. *J. Neuroeng. Rehabil.* 8 1–13. 10.1186/1743-0003-8-34 21682906 PMC3133548

[B59] Hong K. S. Naseer N. Kim Y. H. (2015). Classification of prefrontal and motor cortex signals for three-class fNIRS–BCI. *Neurosci. Lett.* 587 87–92. 10.1016/j.neulet.2014.12.029 25529197

[B60] Hromádka T. DeWeese M. R. Zador A. M. (2008). Sparse representation of sounds in the unanesthetized auditory cortex. *PLoS Biol.* 6:e16. 10.1371/journal.pbio.0060016 18232737 PMC2214813

[B61] Hwang H. Lim J. Kim D. Im C. (2014). Evaluation of various mental task combinations for near-infrared spectroscopy-based brain-computer interfaces. *J. Biomed. Opt.* 19:077005. 10.1117/1.JBO.19.7.077005 25036216

[B62] Ito I. Ong R. C. Raman B. Stopfer M. (2008). Sparse odor representation and olfactory learning. *Nat. Neurosci.* 11 1177–1184. 18794840 10.1038/nn.2192 PMC3124899

[B63] Jang S. J. Yang Y. J. Ryun S. Kim J. S. Chung C. K. Jeong J. (2022). Decoding trajectories of imagined hand movement using electrocorticograms for brain–machine interface. *J. Neural Eng.* 19:056011. 10.1088/1741-2552/ac8b37 35985293

[B64] Johnson K. O. (2000). Neural coding. *Neuron* 26 563–566. 10896153 10.1016/s0896-6273(00)81193-9

[B65] Jolivet R. Rauch A. Lüscher H. Gerstner W. (2006). Predicting spike timing of neocortical pyramidal neurons by simple threshold models. *J. Comput. Neurosci.* 21 35–49. 10.1007/s10827-006-7074-5 16633938

[B66] Kaiser V. Bauernfeind G. Kreilinger A. Kaufmann T. Kübler A. Neuper C. (2014). Cortical effects of user training in a motor imagery based brain–computer interface measured by fNIRS and EEG. *Neuroimage* 85 432–444. 10.1016/j.neuroimage.2013.04.097 23651839

[B67] Kandel E. R. Koester J. D. Sarah H. Mack S. H. Siegelbaum S. A. (2000). *Principles of neural science.* New York, NY: McGraw-Hill.

[B68] Kanerva P. (1988). *Sparse distributed memory.* Cambridge, CA: MIT Press.

[B69] Katzner S. Nauhaus I. Benucci A. Bonin V. Ringach D. L. Carandini M. (2009). Local origin of field potentials in visual cortex. *Neuron* 61 35–41. 19146811 10.1016/j.neuron.2008.11.016 PMC2730490

[B70] Kennedy P. R. Kirby M. T. Moore M. M. King B. Mallory A. (2004). Computer control using human intracortical local field potentials. *IEEE Trans. Neural Syst. Rehabil. Eng.* 12 339–344. 15473196 10.1109/TNSRE.2004.834629

[B71] Klobassa D. S. Vaughan T. M. Brunner P. Schwartz N. E. Wolpaw J. R. Neuper C. (2009). Toward a high-throughput auditory P300-based brain–computer interface. *Clin. Neurophysiol.* 120 1252–1261. 10.1016/j.clinph.2009.04.019 19574091 PMC2729552

[B72] Kostal L. Lansky P. Rospars J. P. (2007). Neuronal coding and spiking randomness. *Eur. J. Neurosci.* 26 2693–2701. 18001270 10.1111/j.1460-9568.2007.05880.x

[B73] Kubánek J. Miller K. J. Ojemann J. G. Wolpaw J. R. Schalk G. (2009). Decoding flexion of individual fingers using electrocorticographic signals in humans. *J. Neural Eng.* 6:066001. 10.1088/1741-2560/6/6/066001 PMC3664231 19794237

[B74] Kübler A. (2020). The history of BCI: From a vision for the future to real support for personhood in people with locked-in syndrome. *Neuroethics* 13 163–180.

[B75] Kübler A. Holz E. M. Riccio A. Zickler C. Kaufmann T. Kleih S. C. (2014). The user-centered design as novel perspective for evaluating the usability of BCI-controlled applications. *PLoS One* 9:e112392. 10.1371/journal.pone.0112392 25469774 PMC4254291

[B76] Kübler A. Nijboer F. Kleih S. (2020). Hearing the needs of clinical users. *Handb. Clin. Neurol.* 168 353–368. 32164866 10.1016/B978-0-444-63934-9.00026-3

[B77] Kübler A. Zickler C. Holz E. Kaufmann T. Riccio A. Mattia D. (2013). Applying the user-centred design to evaluation of Brain-Computer Interface controlled applications. *Biomed. Eng*. 58, 3234–3234. 10.1515/bmt-2013-4438 24043192

[B78] Lee H. Battle A. Raina R. Ng A. (2006). “Efficient sparse coding algorithms,” in *Proceeding of the advances in neural information processing systems*, (Cambridge, MA: MIT Press), 19.

[B79] Li Z. Zhang S. Pan J. (2019). Advances in hybrid brain-computer interfaces: Principles, design, and applications. *Comput. Intell. Neurosci*. 2019. 10.1155/2019/3807670 PMC6800963 31687006

[B80] Liberati G. Pizzimenti A. Simione L. Riccio A. Schettini F. Inghilleri M. (2015). Developing brain-computer interfaces from a user-centered perspective: Assessing the needs of persons with amyotrophic lateral sclerosis, caregivers, and professionals. *Appl. Ergon.* 50 139–146. 10.1016/j.apergo.2015.03.012 25959328

[B81] Logothetis N. K. Pauls J. Augath M. Trinath T. Oeltermann A. (2001). Neurophysiological investigation of the basis of the fMRI signal. *Nature* 412 150–157. 11449264 10.1038/35084005

[B82] Lotte F. Bougrain L. Cichocki A. Clerc M. Congedo M. Rakotomamonjy A. (2018). A review of classification algorithms for EEG-based brain–computer interfaces: A 10 year update. *J. Neural Eng.* 15 031005. 10.1088/1741-2552/aab2f2 29488902

[B83] Lotte F. Congedo M. Anatole L. Lamarche F. Arnaldi B. (2007). A review of classification algorithms for EEG-based brain–computer interfaces. *J. Neural Eng.* 4 R1–R13. 10.1088/1741-2560/4/2/R01 17409472

[B84] Luo S. Rabbani Q. Crone N. E. (2022). Brain-computer interface: Applications to speech decoding and synthesis to augment communication. *Neurotherapeutics* 19 263–273. 10.1007/s13311-022-01190-2 35099768 PMC9130409

[B85] Lyu X. Ding P. Li S. Dong Y. Su L. Zhao L. (2023). Human factors engineering of BCI: An evaluation for satisfaction of BCI based on motor imagery. *Cogn. Neurodyn.* 17 105–118. 36704636 10.1007/s11571-022-09808-z PMC9871150

[B86] Martin S. Armstrong E. Thomson E. Vargiu E. Sol M. Dauwalder S. (2018). A qualitative study adopting a user-centered approach to design and validate a brain computer interface for cognitive rehabilitation for people with brain injury. *Assist. Technol.* 30 233–241. 10.1080/10400435.2017.1317675 28708963

[B87] Mathis A. Herz A. V. Stemmler M. B. (2012). Resolution of nested neuronal representations can be exponential in the number of neurons. *Phys. Rev. Lett.* 109:018103. 10.1103/PhysRevLett.109.018103 23031134

[B88] Maunsell J. H. Van Essen D. C. (1983). Functional properties of neurons in middle temporal visual area of the macaque monkey. I. Selectivity for stimulus direction, speed, and orientation. *J. Neurophysiol.* 49 1127–1147. 6864242 10.1152/jn.1983.49.5.1127

[B89] McMillan G. R. Calhoun G. L. Middendorf M. S. Schnurer J. Ingle D. Nasman V. (1995). “Direct brain interface utilizing self-regulation of steady-state visual evoked response (SSVER),” in *Proceedings of the RESNA ‘95 Annual Conference*, (Vancouver, BC), 693–695.

[B90] Mehler D. M. Sokunbi M. O. Habes I. Barawi K. Subramanian L. Range M. (2018). Targeting the affective brain—a randomized controlled trial of real-time fMRI neurofeedback in patients with depression. *Neuropsychopharmacology* 43 2578–2585. 10.1038/s41386-018-0126-5 29967368 PMC6186421

[B91] Mellinger J. Schalk G. Braun C. Preissl H. Rosenstiel W. Birbaumer N. (2007). An MEG-based brain–computer interface (BCI). *Neuroimage* 36 581–593. 17475511 10.1016/j.neuroimage.2007.03.019 PMC2017111

[B92] Metzger S. L. Littlejohn K. T. Silva A. B. Moses D. A. Seaton M. P. Wang R. (2023). A high-performance neuroprosthesis for speech decoding and avatar control. *Nature* 620 1037–1046. 10.1038/s41586-023-06443-4 37612505 PMC10826467

[B93] Milekovic T. Bacher D. Sarma A. A. Simeral J. D. Saab J. Pandarinath C. (2019). Volitional control of single-electrode high gamma local field potentials by people with paralysis. *J. Neurophysiol.* 121 1428–1450. 10.1152/jn.00131.2018 30785814 PMC6485734

[B94] Milekovic T. Sarma A. A. Bacher D. Simeral J. D. Saab J. Pandarinath C. (2018). Stable long-term BCI-enabled communication in ALS and locked-in syndrome using LFP signals. *J. Neurophysiol.* 120 343–360. 10.1152/jn.00493.2017 29694279 PMC6093965

[B95] Miller K. J. Leuthardt E. C. Schalk G. Rao R. P. Anderson N. R. Moran D. W. (2007). Spectral changes in cortical surface potentials during motor movement. *J. Neurosci.* 27 2424–2432. 17329441 10.1523/JNEUROSCI.3886-06.2007 PMC6673496

[B96] Miller K. J. Schalk G. Hermes D. Ojemann J. G. Rao R. P. (2016). Spontaneous decoding of the timing and content of human object perception from cortical surface recordings reveals complementary information in the event-related potential and broadband spectral change. *PLoS Comput. Biol.* 12:e1004660. 10.1371/journal.pcbi.1004660 PMC4731148 26820899

[B97] Miller K. J. Zanos S. Fetz E. E. Nijs M d Ojemann J. G. (2009). Decoupling the cortical power spectrum reveals real-time representation of individual finger movements in humans. *J. Neurosci.* 29 3132–3137. 10.1523/JNEUROSCI.5506-08.2009 19279250 PMC6666461

[B98] Miller M. I. Sachs M. B. (1983). Representation of stop consonants in the discharge patterns of auditory-nerve fibers. *J. Acoust. Soc. Am.* 74 502–517. 6619427 10.1121/1.389816

[B99] Ming C. Shangkai G. (1999). “An EEG-based cursor control system,” in *Proceedings of the 1999 IEEE engineering in medicine and biology 21st annual conference and the 1999 annual fall meeting of the biomedical engineering society*, (Piscataway, NJ).

[B100] Molina G. G. Mihajlovic V. (2010). Spatial filters to detect steady-state visual evoked potentials elicited by high frequency stimulation: BCI application. *Biomed. Tech.* 55 173–182. 10.1515/BMT.2010.013 20415628

[B101] Montemurro M. A. Rasch M. J. Murayama Y. Logothetis N. K. Panzeri S. (2008). Phase-of-firing coding of natural visual stimuli in primary visual cortex. *Curr. Biol.* 18 375–380. 10.1016/j.cub.2008.02.023 18328702

[B102] Monti M. M. Vanhaudenhuyse A. Coleman M. R. Boly M. Pickard J. D. Tshibanda L. (2010). Willful modulation of brain activity in disorders of consciousness. *N. Engl. J. Med.* 362 579–589. 20130250 10.1056/NEJMoa0905370

[B103] Müller S. M. Diez P. F. Bastos-Filho T. F. Sarcinelli-Filho M. Mut V. Laciar E. (2011). “SSVEP-BCI implementation for 37–40 Hz frequency range,” in *Proceedings of the 2011 annual international conference of the IEEE engineering in medicine and biology society*, (Piscataway, NJ), 6352–6355. 10.1109/IEMBS.2011.6091568 22255791

[B104] Müller-Putz G. R. Scherer R. Neuper C. Pfurtscheller G. (2006). Steady-state somatosensory evoked potentials: Suitable brain signals for brain-computer interfaces? *IEEE Trans. Neural Syst. Rehabil. Eng.* 14 30–37. 10.1109/TNSRE.2005.863842 16562629

[B105] Müller-Putz G. R. Zimmermann D. Graimann B. Nestinger K. Korisek G. Pfurtscheller G. (2007). Event-related beta EEG-changes during passive and attempted foot movements in paraplegic patients. *Brain Res.* 1137 84–91. 10.1016/j.brainres.2006.12.052 17229403

[B106] Mussi M. G. Adams K. D. (2022). EEG hybrid brain-computer interfaces: A scoping review applying an existing hybrid-BCI taxonomy and considerations for pediatric applications. *Front. Hum. Neurosci.* 16:1007136. 10.3389/fnhum.2022 PMC9715435 36466619

[B107] Naseer N. Hong M. J. Hong K. S. (2014). Online binary decision decoding using functional near-infrared spectroscopy for the development of brain–computer interface. *Exp. Brain Res.* 232 555–564. 10.1007/s00221-013-3764-1 24258529

[B108] Naselaris T. Kay K. N. Nishimoto S. Gallant J. L. (2011). Encoding and decoding in fMRI. *Neuroimage* 56 400–410. 20691790 10.1016/j.neuroimage.2010.07.073 PMC3037423

[B109] Needell D. Tropp J. A. (2009). CoSaMP: Iterative signal recovery from incomplete and inaccurate samples. *Appl. Comput. Harmon. Anal.* 26 301–321.

[B110] Neuper C. Scherer R. Wriessnegger S. Pfurtscheller G. (2009). Motor imagery and action observation: Modulation of sensorimotor brain rhythms during mental control of a brain–computer interface. *Clin. Neurophysiol.* 120 239–247. 10.1016/j.clinph.2008.11.015 19121977

[B111] Ogawa S. Lee T. M. Kay A. R. Tank D. W. (1990). Brain magnetic resonance imaging with contrast dependent on blood oxygenation. *Proc. Natl. Acad. Sci. U.S.A.* 87 9868–9872. 2124706 10.1073/pnas.87.24.9868 PMC55275

[B112] Olshausen B. A. Field D. J. (1996). Emergence of simple-cell receptive field properties by learning a sparse code for natural images. *Nature* 381 607–609. 10.1038/381607a0 8637596

[B113] Oxley T. J. Opie N. L. John S. E. Rind G. S. Ronayne S. M. Wheeler T. L. (2016). Minimally invasive endovascular stent-electrode array for high-fidelity, chronic recordings of cortical neural activity. *Nat. Biotechnol.* 34 320–327. 10.1038/nbt.3428 26854476

[B114] Oxley T. J. Yoo P. E. Rind G. S. Ronayne S. M. Lee C. M. Bird C. (2021). Motor neuroprosthesis implanted with neurointerventional surgery improves capacity for activities of daily living tasks in severe paralysis: First in-human experience. *J. Neurointerv. Surg.* 13 102–108. 33115813 10.1136/neurintsurg-2020-016862 PMC7848062

[B115] Panzeri S. Schultz S. R. Treves A. Rolls E. T. (1999). Correlations and the encoding of information in the nervous system. *Proc. R. Soc. Lond. Ser. B Biol. Sci.* 266 1001–1012. 10.1098/rspb.1999.0736 PMC1689940 10610508

[B116] Pati Y. C. Rezaiifar R. Krishnaprasad P. S. (1993). “Orthogonal matching pursuit: Recursive function approximation with applications to wavelet decomposition,” in *Proceedings of 27th Asilomar conference on signals, systems and computers*, (Piscataway, NJ), 40–44.

[B117] Paulmurugan K. Vijayaragavan V. Ghosh S. Padmanabhan P. Gulyás B. (2021). Brain–computer interfacing using functional near-infrared spectroscopy (fNIRS). *Biosensors* 11:389. 10.3390/bios11100389 PMC8534036 34677345

[B118] Pfurtscheller G. Graimann B. Huggins J. E. Levine S. P. Schuh L. A. (2003a). Spatiotemporal patterns of beta desynchronization and gamma synchronization in corticographic data during self-paced movement. *Clin. Neurophysiol.* 114 1226–1236. 10.1016/s1388-2457(03)00067-1 12842719

[B119] Pfurtscheller G. Müller G. R. Pfurtscheller J. Gerner H. J. Rupp R. (2003b). ‘Thought’–control of functional electrical stimulation to restore hand grasp in a patient with tetraplegia. *Neurosci. Lett.* 351 33–36. 10.1016/s0304-3940(03)00947-9 14550907

[B120] Pfurtscheller G. Neuper C. Flotzinger D. Pregenzer M. (1997). EEG-based discrimination between imagination of right and left hand movement. *Electroencephalogr. Clin. Neurophysiol.* 103 642–651. 9546492 10.1016/s0013-4694(97)00080-1

[B121] Ramoser H. Muller-Gerking J. Pfurtscheller G. (2000). Optimal spatial filtering of single trial EEG during imagined hand movement. *IEEE Trans. Rehabil. Eng.* 8 441–446. 11204034 10.1109/86.895946

[B122] Ramsey N. Millán José del R. (2020). *Brain-computer interfaces.* Amsterdam: Elsevier.

[B123] Rathee D. Raza H. Roy S. Prasad G. (2021). A magnetoencephalography dataset for motor and cognitive imagery-based brain-computer interface. *Sci. Data* 8:120. 10.1038/s41597-021-00899-7 33927204 PMC8085139

[B124] Rehn M. Sommer F. T. (2007). A network that uses few active neurones to code visual input predicts the diverse shapes of cortical receptive fields. *J. Comput. Neurosci.* 22 135–146. 10.1007/s10827-006-0003-9 17053994

[B125] Rickert J. Oliveira S. C. Vaadia E. Aertsen A. Rotter S. Mehring C. (2005). Encoding of movement direction in different frequency ranges of motor cortical local field potentials. *J. Neurosci.* 25 8815–8824. 16192371 10.1523/JNEUROSCI.0816-05.2005 PMC6725584

[B126] Seitz R. J. (2010). How imaging will guide rehabilitation. *Curr. Opin. Neurol.* 23 79–86. 19926990 10.1097/WCO.0b013e328334c84d

[B127] Senden M. Emmerling T. C. Hoof R. Frost M. A. Goebel R. (2019). Reconstructing imagined letters from early visual cortex reveals tight topographic correspondence between visual mental imagery and perception. *Brain Struct. Funct.* 224 1167–1183. 10.1007/s00429-019-01828-6 30637491 PMC6499877

[B128] Shirhatti V. Borthakur A. Ray S. (2016). Effect of reference scheme on power and phase of the local field potential. *Neural Comput.* 28 882–913. 10.1162/NECO_a_00827 26942748 PMC7117962

[B129] Siero J. C. Hermes D. Hoogduin H. Luijten P. R. Petridou N. Ramsey N. F. (2013). BOLD consistently matches electrophysiology in human sensorimotor cortex at increasing movement rates: A combined 7T fMRI and ECoG study on neurovascular coupling. *J. Cereb. Blood Flow Metab.* 33 1448–1456. 10.1038/jcbfm.2013.97 23801242 PMC3764395

[B130] Siero J. C. Hermes D. Hoogduin H. Luijten P. R. Ramsey N. F. Petridou N. (2014). BOLD matches neuronal activity at the mm scale: A combined 7 T fMRI and ECoG study in human sensorimotor cortex. *Neuroimage* 101 177–184. 10.1016/j.neuroimage.2014.07.002 25026157

[B131] Sorger B. Reithler J. Dahmen B. Goebel R. (2012). A real-time fMRI-based spelling device immediately enabling robust motor-independent communication. *Curr. Biol.* 22 1333–1338. 10.1016/j.cub.2012.05.022 22748322

[B132] Sun Y. Shen A. Sun J. Du C. Chen X. Wang Y. (2022). Minimally invasive local-skull electrophysiological modification with piezoelectric drill. *IEEE Trans. Neural Syst. Rehabil. Eng.* 30 2042–2051. 10.1109/TNSRE.2022.3192543 35857723

[B133] Theunissen F. Miller J. P. (1995). Temporal encoding in nervous systems: A rigorous definition. *J. Comput. Neurosci.* 2 149–162. 10.1007/BF00961885 8521284

[B134] Thorpe S. J. (1990). “Spike arrival times: A highly efficient coding scheme for neural networks,” in *Parallel processing in neural systems*, eds Hartmann G. Eckmiller R. Hauske G. (North-Holland: Elsevier), 91–94.

[B135] Vansteensel M. J. Pels E. G. Bleichner M. G. Branco M. P. Denison T. Freudenburg Z. V. (2016). Fully implanted brain–computer interface in a locked-in patient with ALS. *N. Engl. J. Med.* 375 2060–2066. 10.1056/NEJMoa1608085 27959736 PMC5326682

[B136] Victor J. D. (2005). Spike train metrics. *Curr. Opin. Neurobiol.* 15 585–592. 16140522 10.1016/j.conb.2005.08.002 PMC2713191

[B137] Vision N. (2000). Sparse coding and decorrelation in primary visual cortex during. *Science* 287:1273. 10.1126/science.287.5456.1273 10678835

[B138] Volosyak I. Valbuena D. Lüth T. Malechka T. Gräser A. (2011). BCI demographics II: How many (and what kinds of) people can use a high-frequency SSVEP BCI? *IEEE Trans. Neural Syst. Rehabil. Eng.* 19 232–239. 10.1109/TNSRE.2011.2121919 21421448

[B139] Wang W. (2006). *Motor cortical representation of hand position, velocity and rotation during reaching.* St. Louis, MO: Washington University.

[B140] Wang Z. Shi N. Zhang Y. Zheng N. Li H. Jiao Y. (2023). Conformal in-ear bioelectronics for visual and auditory brain-computer interfaces. *Nat. Commun.* 14:4213. 10.1038/s41467-023-39814-6 37452047 PMC10349124

[B141] Willett F. R. Avansino D. T. Hochberg L. R. Henderson J. M. Shenoy K. V. (2021). High-performance brain-to-text communication via handwriting. *Nature* 593 249–254. 10.1038/s41586-021-03506-2 33981047 PMC8163299

[B142] Willett F. R. Kunz E. M. Fan C. Avansino D. T. Wilson G. H. Choi E. Y. (2023). A high-performance speech neuroprosthesis. *Nature* 620 1031–1036. 37612500 10.1038/s41586-023-06377-x PMC10468393

[B143] Wittevrongel B. Khachatryan E. Fahimi H. M. Camarrone F. Carrette E. De Taeye L. (2018). Decoding steady-state visual evoked potentials from electrocorticography. *Front. Neuroinform.* 12:65. 10.3389/fninf.2018.00065 30319386 PMC6168710

[B144] Wolpaw J. R. Wolpaw E. W. (2012). *Brain-computer interfaces: Principles and practice.* Oxford: Oxford University Press.

[B145] Wolpaw J. R. Millán J. R. Ramsey N. F. (2020). Brain-computer interfaces: Definitions and principles. *Handb. Clin. Neurol.* 168 15–23. 32164849 10.1016/B978-0-444-63934-9.00002-0

[B146] Wu S. Amari S. Nakahara H. (2002). Population coding and decoding in a neural field: A computational study. *Neural Comput.* 14 999–1026. 10.1162/089976602753633367 11972905

[B147] Xu H. Gong A. Ding P. Luo J. Chen C. Fu Y. (2022). Key technologies for intelligent brain-computer interaction based on magnetoencephalography. *Sheng Wu Yi Xue Gong Cheng Xue Za Zhi* 39 198–206. 10.7507/1001-5515.202108069 35231982 PMC9927744

[B148] Xu L. Xu M. Jung T. P. Ming D. (2021). Review of brain encoding and decoding mechanisms for EEG-based brain–computer interface. *Cogn. Neurodyn.* 15 569–584. 34367361 10.1007/s11571-021-09676-z PMC8286913

[B149] Xue H. Wang D. Jin M. Gao H. Wang X. Xia L. (2023). Hydrogel electrodes with conductive and substrate-adhesive layers for noninvasive long-term EEG acquisition. *Microsyst. Nanoeng.* 9:79. 10.1038/s41378-023-00524-0 37313471 PMC10258200

[B150] Yoo S. S. Choi B. G. Chung K. I. Lee C. (2001). Neural substrates of motor imagery: Event-related functional MRI study. *J. Korean Neuropsychiatr. Assoc.* 1247–1250.

[B151] Yoo S. S. Fairneny T. Chen N. K. Choo S. E. Panych L. P. Park H. (2004). Brain–computer interface using fMRI: Spatial navigation by thoughts. *Neuroreport* 15 1591–1595. 15232289 10.1097/01.wnr.0000133296.39160.fe

[B152] Zhang D. Liu S. Wang K. Zhang J. Chen D. Zhang Y. (2021). Machine-vision fused brain machine interface based on dynamic augmented reality visual stimulation. *J. Neural Eng.* 18:056061. 10.1088/1741-2552/ac2c9e 34607320

[B153] Zhang M. Wu J. Song J. Fu R. Ma R. Jiang Y. (2022). Decoding coordinated directions of bimanual movements from EEG signals. *IEEE Trans. Neural Syst. Rehabil. Eng.* 31 248–259. 10.1109/TNSRE.2022.3220884 36350872

[B154] Zhang Z. Constandinou T. G. (2023). Firing-rate-modulated spike detection and neural decoding co-design. *J. Neural Eng.* 20:036003. 10.1088/1741-2552/accece 37080210

