# Supplementary material for: Brain-computer interface paradigms and neural coding
Source: Front Neurosci. 2024 Jan 15;17:1345961. doi: 10.3389/fnins.2023.1345961 (PMC10822902; doi:10.3389/fnins.2023.1345961)
Supplement: Supplementary file 1 [file Table_4.DOCX]

Supplementary Material

# Supplementary Tables

Tables 1 Examples for existing main LFP-BCI paradigms and neural coding

| References | Paradigms | Neural Coding | Main Conclusions |
| --- | --- | --- | --- |
| Kennedy et al. (2004) [73] | Task 1: controlling the flexion of a biomimetic finger, Task 2: moving the cursor horizontally and vertically | Using LFP amplitude exceeding the threshold to control the flexion of biomimetic fingers | LFP recorded by neural nutrient electrodes implanted in the motor cortex is expected to be used for controlling assistive devices |
| Rickert et al. (2005) [75] | Macaque was trained to follow visual cues and use either of its two arms to perform center-out arm movements to control the movement of two cursors on a screen | Directions of movement were tuned by increased amplitudes of three frequency ranges (<=4, 6-13, and 63-200 Hz) of LFP within motor cortex | Directions of movement can be decoded using the amplitudes of different frequency components of LFP, and different combinations of frequency bands can be used to get the best performance |
| Heldman et al, (2006) [76] | Macaque was trained to follow visual cues and perform a standard three-dimensional (3D) center-out reaching task | Velocities were encoded by about 18 % of the high frequency bands (HFB) (60 - 200 Hz) LFP within the motor cortex and positions were encoded by 15 % of the HF-LFP | Controlling multiple degrees of freedom by BCI based on HFB-LFP recorded within motor cortex |
| Wang et al, (2007) [77] | Macaque was trained to perform a center-out reaching task in a virtual reality environment | Hand location and velocity were encoded by HFB-LFP (60-200 Hz) power spectrum amplitudes and neuronal activity of single unit within the motor cortex | Simultaneous decoding of three-dimensional hand position and velocity from relevant neural activities |
| Milekovic et al, (2018) [78] | Subjects attempted to perform the selected action to choose an option | Movements were encoded by HFB-LFP of single unit activity recorded from microelectrodes implanted in the motor cortex | Microelectrodes implanted in subjects for long periods continue to provide high-fidelity signals that can achieve precise BCI control |
| Milekovic et al. (2019) [79] | Subjects with tetraplegia performed real or/and imagined movements to control a one-dimensional cursor moving up and down | Single-channel HFB-LFP (40-400 Hz) recorded within the motor cortex could be modulated by neurofeedback and encode motor intentions | LFP modulated by neurofeedback may contribute to reliable control of actuators |
| Ahmadi et al.（2021）[80] | Training monkeys to respond to visual cues, they perform point-to-point tasks by moving their fingertips to randomly appearing circular targets | The local motion potential (LMP) in the temporal domain of the primary motor cortex's local field potentials (LFP) exhibits more significant encoding of motor intent compared to frequency domain features (delta, theta, alpha, beta, and gamma bands) | Combining LMP with a common average reference can enhance the decoding performance of LFP-based Brain-Computer Interfaces (BCI) |
| Zhang et al.（2023） [81] | Training monkeys to respond to visual cues, they perform point-to-point tasks by moving their fingertips to randomly appearing circular targets | In the primary motor cortex, LFP at a fixed threshold of 46 Hz significantly encodes motor intent | The use of threshold settings based on peak detection effectively manages the trade-off between data bandwidth and decoding performance, without relying on training or trial and error |
