# Supplementary material for: Brain-computer interface paradigms and neural coding
Source: Front Neurosci. 2024 Jan 15;17:1345961. doi: 10.3389/fnins.2023.1345961 (PMC10822902; doi:10.3389/fnins.2023.1345961)
Supplement: Supplementary file 2 [file Table_5.DOCX]

Supplementary Material

# Supplementary Tables

Table 2 Examples for existing main ECoG-BCI paradigms and neural coding research

| References | Paradigms | Neural Coding | Main Conclusions |
| --- | --- | --- | --- |
| Kubanek et al. (2009) [85] | Subjects were asked to flex a specific finger contralateral to the ECoG electrode on cue | Localized ECoG motor potentials in sensorimotor areas combined with specific frequency band (75-115 Hz) signals encode specific finger flexion | ECoG signals can accurately infer repetitive and rhythmic finger flexion in humans |
| Brunner et al. (2011) [86] | The subject's task was to note the characters expected in the 6 × 6 P300 matrix speller | ERPs in visual areas significantly encoded subjects' desired characters | With the P300 Matrix Speller, ECoG enables faster communication speeds than EEG |
| Gunduz et al. (2012) [87] | Subjects were asked to focus their attention on the stimulus location indicated by the arrow and press the key in response to the direction of the cue | Frontal-parietal ECoG features of alpha, beta, and high gamma spectral amplitude changes encode shifts and locations of subjects' covert attention | Using ECoG signals is expected to decode human covert spatial attention shifts and locations |
| Miller et al. (2016) [88] | Subjects focused on gray pictures of faces and houses | Surface evoked potentials (N200) and broadband power changes in the ventral temporal cortex encode the timing of stimulus appearance and image type | Simultaneous use of broadband response and ERP allows prediction of stimulus onset time and image type |
| Burke et al. (2013) [89], (2014) [90] | A list of words is presented to the subject and after a distraction interval, the subject is asked to verbally recall as many words as possible in any order | The specific frequency band of theta (4-8 Hz and alpha (9-14 Hz) power changes in the left frontal and medial and lateral time lobes characterize memory formation processes | Electrophysiological signals may be causally related to specific behavioral conditions and episodic stimulus presentation has the potential to modulate human memory encoding |
| Hermes et al. (2015) [91] | Subjects focused on a series of static images of grids and noise patterns | Narrowband gamma band (30-80 Hz) oscillations in visual cortex (V1/V2/V3) encoded grating pattern stimuli; broadband gamma band (80-200 Hz) power spectral changes characterized noise pattern stimuli | Spatial contrast raster pattern stimuli reliably induce narrowband gamma oscillations that are not induced by noisy patterns and many natural images; all visual stimuli induce broadband gamma power spectrum changes |
| Hermes et al. (2017) [92] | Subjects gazed at different grating stimuli of decreasing the size or contrast of the grating, superimposing a second grating to produce a checkerboard grid, superimposing noise, etc. | Different grating stimuli can be characterized by varying degrees of narrowband gamma (30 to 80 Hz) oscillations and broadband gamma (80-200 Hz) power spectral changes in the visual cortex | Reducing the size or contrast of the grating stimulus, superimposing a second grating to create a checkerboard grid or superimposing noise can reduce the light burst response |
| Vansteensel et al. (2016) [93] | subject tries to move the right hand to move the cursor up, then relaxes the hand to move the cursor down to select the target on the screen | High-frequency broadband power changes in ECoG in hand areas and left prefrontal areas of the left motor cortex characterize hand movement and relaxation | Characterization of high-frequency broadband power changes in ECoG recorded on the motor cortex can be used to control spelling devices |
| Hermes et al. (2011) [94] | Subjects perform or imagine movement that alternately tapping the thumb with the right or left fingers or being in a resting state, at the required tempo | ECoG low-frequency (8-24 Hz) power reduction and high-frequency (65-95 Hz) power increase in the premotor cortex during performed or imagined thumb tapping | Brain regions other than primary motor areas can be more reliably activated during motor imagery, and the premotor cortex may be a better brain region for implantable BCIs |
| Hermes et al. (2012) [95] | Subjects were asked to close and open their right hand at four different rates (0.3, 0.5, 1, and 2 Hz). | ECoG high-frequency power (65-95 Hz) in the motor and sensory cortex was significantly suppressed during faster rates of movement (1, 2 Hz), and beta power between movements did not return to baseline and remained suppressed. the magnitude of beta band suppression did not change with different rates | Recent exercise affects the state of the motor cortex and facilitates the next exercise by reducing the level of neuronal activity required |
| Hermes et al. (2014) [96] | The patient performs a covert verb generation task in which, based on the prompted noun, the patient must covertly think of a matching verb | Verb generation processes can be characterized by significant decreases in theta (4-7 Hz) power in Broca's area and temporal lobe speech areas | There is a dynamic interaction between the neuronal mechanisms of θ rhythms and localized high-frequency (65-95 Hz) neuronal activity in the language area, which shows a marked attenuation of θ rhythms |
| Siero et al. (2013) [97] | Subjects were asked to close and open their right hand at four different rates (about 0.3, 0.5, 1, and 2 Hz) in visual cues | BOLD saturation at different rates of movement can be explained by a decrease in HFB power in primary motor cortex (M1) and primary sensory cortex (S1) | In sensorimotor cortex, a large portion of the BOLD nonlinearity related to movement rate can be well predicted by electrophysiology |
| Siero et al. (2014) [98] | Subjects performed thumb, index, or little finger flexion movements following visual cues | The BOLD signal of 7T and ECoG HFB power changes are closely correlated, and both are able to discriminate between neuronal population activity patterns activated by individual finger movements on a 1-mm meter scale | In sensorimotor cortical areas, the spatial distribution of BOLD signaling activation matches the spatial distribution of ECoG HFB power changes |
| Miller et al. (2009) [99]. | Subjects moved their fingers independently during a 2-second movement trial in a visual cue | Frequency wideband (5-200 Hz) power changes show spatially discrete representations of individual fingers and reproduce temporal trajectories of different finger movements | Decoupling of cortical power spectra reveals a real-time representation of human finger movements |
| Crone et al., (1998) [100] | Subjects were asked to make sustained isometric muscle contractions in different parts of the body in response to a visual cue (extending the tongue, clenching the fist, or dorsiflexing the foot) | α-ERDs and β-ERDs are associated with cortical activation, with α-ERDs having a high degree of variability, and β-ERDs may have higher specificity and lower sensitivity to cortical activation, a more rapid response, and a greater tendency to rebound compared with α-ERDs | Unilateral limb movements produce sustained α-ERDs and β-ERDs in the bilateral sensorimotor cortex, with overlapping patterns in different body parts |
| Miller et al. (2007) [101] | Subjects performed simple, repetitive hand movements and tongue movements | HFB (76-100 Hz) and Low Frequency Band (LFB) (8-32 Hz) characterize motion patterns | The LFB (8 -32 Hz) power of the sensorimotor cortex continued to decrease with movement, whereas the HFB (76 -100 Hz) power continued to increase. changes in the HFB were more focused than changes in the LFB |
| Pfurtscheller et al. (2003) [102] | Subjects performed repetitive movements of hand movements and tongue movements in a self-paced manner | Time-space features of β-ERD and γ(60 -90 Hz) ERS in ECoG data characterize self-paced motions | Autorhythmic movements are accompanied not only by relatively widely distributed μ and β-ERDs but also by more focused γ-ERS in the 60-90 Hz band |
| Brinkman et al. （2014） [103] | Subjects imagined grasping cylinders at different angles | Grasping motor imagery can be characterized by a concomitant increase in power in the alpha (8-12 Hz) band of the sensory-motor cortex ipsilateral to the imagined arm and a concomitant decrease in power in the beta (15-25 Hz) band of the contralateral sensory-motor cortex | Reduction of beta-band neural oscillations is directly related to the disinhibition of neuronal populations involved in the calculation of motor parameters |
| Brinkman et al. 2016  [104] | AC transcranial stimulation (tACS) was applied at a frequency of 10 or 20 Hz to the sensorimotor cortex contralateral or ipsilateral to the grasping hand while the subject chose how to grasp a cylinder | Functionally distinguishable causal contributions to α (8-12 Hz) band and β (15-25 Hz) band oscillations during movement selection | Beta-band rhythms in sensorimotor areas disinhibit task-related neuronal populations, whereas alpha-band rhythms inhibit neuronal populations that may interfere with motor selection |
| Benjamin et al. (2018) [105] | Subjects were asked to gaze at a flicker stimulus target consisting of different frequency-phase combinations | ECoG-SSVEP recorded in the right occipital cortex encoded stimuli of different frequencies and/or phase | ECoG-based decoding is more accurate for very short stimulus lengths ( that is, less than 1 second), and fewer ECoG electrodes produce faster decoding compared to EEG-SSVEP |
| Sang Jin Jang et al.（2022）[106] | Subjects perform continuous stages of reaching and grasping actions based on visual and auditory cues ("start," "reach," "grasp," "return," and "release") | The trajectory of imagined hand movements can be encoded based on the optimal electrodes, frequencies, and time lags in electrocorticography (ECoG) | The paradigm of alternating trials between motor execution (ME) and kinesthetic motor imagery (KMI) demonstrates higher decoding accuracy for imagined hand movement trajectories compared to the isolated KMI paradigm. |
