# Supplementary material for: Brain-computer interface paradigms and neural coding
Source: Front Neurosci. 2024 Jan 15;17:1345961. doi: 10.3389/fnins.2023.1345961 (PMC10822902; doi:10.3389/fnins.2023.1345961)
Supplement: Supplementary file 3 [file Table_6.DOCX]

Supplementary Material

# Supplementary Tables

Tables 3 Examples for existing main fMRI -BCI paradigms and neural coding

| References | Paradigms | Neural Coding | Main Conclusions |
| --- | --- | --- | --- |
| Yoo et al. (2003) [111] | Subjects with a healthy right hand imagined clenching their right hand when prompted by auditory stimuli | Bilateral primary, premotor, and supplementary motor areas showed MRI signal changes associated with imagined events during imagined clenching of the right hand | Motor imagery and actual movement have a common neural substrate |
| Yoo et al. (2004) [112] | Subjects completed tasks of simple mental calculations, covert speech production, and fist-shaking motor imagery | Bilateral activation of the dorsolateral prefrontal cortex characterized mental computation; the left inferior frontal gyrus and auditory association area characterized covert speech tasks; and left/right somatosensory-motor areas respectively characterized right-handed imagery and left-handed imagery | The spatial distribution of activation characterized by real-time fMRI can be used as a source signal for BCI |
| Boly et al. (2007) [113] | Task 1: Alternate among space navigation imagery, silent retelling, and rest; Task 2: Alternate among imagery of playing tennis, facial imagery, and rest | Bilateral precuneus/parieto-occipital junction and post-pressor cortex activation characterized spatial navigation, with left superior temporal gyrus activated by silent repetition in only 3/12 subjects; bilateral SMA and inferior parietal lobule activation characterized complex motor imagery tasks (such as imagery of playing tennis), and there was greater variability in cortical activation of facial imagery between subjects | Tasks of space navigation imagery and complex motor imagery allow the identification of brain activation for intentional imagery at the subject level and may provide a method for assessing volitional brain activity as well as the presence of consciousness in patients with non-communicative brain injuries |
| Monti et al. (2010) [114] | Subjects were asked to imagery of playing tennis and space navigation imagery | In a small group of patients in a vegetative or minimally conscious state, tennis imagery elicited extensive activation of supplementary motor areas and minimal activation of the para hippocampal gyrus. Imagery of spatial navigation elicited extensive activation of the Para hippocampal gyrus and minor activation of supplementary motor areas. | A small group of vegetative or a vegetative or minimally conscious states have brain activation during the performance of imagery tasks, reflecting some awareness and cognition that can reclassify their state of consciousness and basic communication can be established |
| Sorger et al. (2012) [115] | Subjects performed the corresponding mental tasks (motor imagery, mental arithmetic, and inner speech) according to the onset delays (0/10/20s) and durations (10/20/30s) of mental activities corresponding to the desired characters | Spatiotemporal characterization of hemodynamic responses can encode mental imagery tasks with different onset delays and durations, allowing the conversion of desired letters into reliable and distinguishable single-trial fMRI signals | Subjects are able to communicate any given idea in a motion-independent manner in fMRI experiments using novel, robust, and immediately applicable alphabetic coding techniques and advanced real-time fMRI decoding methods |
| Senden et al. (2019) [116] | Subjects were asked to perform visual imagery of four different letter shapes | For each imagined letter (H, T, S, C) ROI (V1, V2, V3) combination, imaginal voxel-activated retinal tissue is geometrically specific enough to distinguish between different imaginal letters | The autoencoder can project imagery-related voxel activation onto its perceptual counterpart, enabling reconstruction of visual recognition at the single-trial level, and can be used to develop a content-based BCI letter speller system. |
