# Supplementary material for: Brain-computer interface paradigms and neural coding
Source: Front Neurosci. 2024 Jan 15;17:1345961. doi: 10.3389/fnins.2023.1345961 (PMC10822902; doi:10.3389/fnins.2023.1345961)
Supplement: Supplementary file 4 [file Table_7.DOCX]

Supplementary Material

# Supplementary Tables

Table 4 Examples for existing main fNIRS -BCI paradigms and neural coding

| References | Paradigms | Neural Coding | Main Conclusions |
| --- | --- | --- | --- |
| Coyle et al. (2007) [117] | Subjects were asked to imagine grasping a ball in their right hand | Significant increase in HbO concentration in the motor area during motor imagery | Subjects communicate with external devices through thought processes ("on/off") |
| Holper et al. (2011) [118] | Subjects were asked to image the right thumb performing a tapping task, or to image all the fingers of the right hand performing a continuous finger-tapping task | Simple combinations of fNIRS channels, time intervals, and four Δ[O2Hb] signal features (including signal amplitude mean, variance, skewness, and kurtosis) in the secondary motion region can characterize motion imagery tasks of varying complexity | Single-trial fNIRS-BCI can categorize motor imagery tasks of varying complexity |
| Kaiser et al. (2014) [119] | Subjects were asked to imagine right hand and foot movements based on arrow cues | Sensory-motor areas C3, C4, and Cz positions showed a significantly enhanced HbO activation pattern | Training subjects with MI-BCI affects their cortical activation patterns, especially in subjects with lower BCI performance |
| Hwang et al. (2014) [120] | Subjects were asked to perform one of eight mental tasks (left/right hand/foot movement imagery, mental singing, mental subtraction/multiplication, imagining geometric rotations, mental writing) based on randomized visual cues | HbO responses during right-handed motor imagery and imagining geometric figure rotations showed similar patterns in frontal regions, but differences between the two tasks increased in parieto-occipital regions. Around the posterior regions of the brain, the HbO responses during imagining geometric figure rotation and mental multiplication were more similar. The HbR responses for the last three mental tasks did not show any consistent spatial pattern | Mental task combinations with relatively high categorization accuracy typically include mental multiplication, mental rotation, and right-handed motor imagery, with two-by-two combinations having the highest average categorization accuracies |
| Hong et al. (2015) [121] | Subjects were asked to perform mental arithmetic, imagery of left/right hand squeezing the ball based on visual cues | The difference between HBO signals collected in the prefrontal and motor cortex during the mental arithmetic and left/right-hand motor imagery tasks and resting tasks can characterize these three types of mental tasks | HbO signals 2-7 s after mental arithmetic, left/right-hand motor imagery tasks are well separable for fNIRS-BCI |
| Naseer et al. (2014) [122] | Subjects were asked to make choices based on the questions and were asked to perform mental arithmetic when making a "yes" decision, while they were asked to relax when making a "no" decision | Using the mean of changes in prefrontal cortical hemoglobin concentration as a feature to characterize binary decision-making "yes" and "no" categories | f fNIRS signaling in the prefrontal cortex verifies that the cortical hemodynamic response to a "yes" decision is different from that to a "no" decision |
