# Supplementary material for: Brain-computer interface paradigms and neural coding
Source: Front Neurosci. 2024 Jan 15;17:1345961. doi: 10.3389/fnins.2023.1345961 (PMC10822902; doi:10.3389/fnins.2023.1345961)
Supplement: Supplementary file 5 [file Table_8.DOCX]

Supplementary Material

# Supplementary Tables

Table 5 Examples for existing main MEG -BCI paradigms and neural coding research

| References |  | | Paradigms | Neural Coding | | Main Conclusions |
| --- | --- | --- | --- | --- | --- | --- |
| Mellinger et al. (2007) [124] | | Subjects were asked to perform actual repetitive hand or foot movements followed by corresponding imagery (left hand vs. right hand, both hands vs. both feet) | | | Motor cortex MEG μ-band ((9-15 Hz) or β-band (18-30 Hz)) power characterizes left-handed versus right-handed or two-handed versus two-footed movement imagery | Subjects achieved significant sensorimotor μ-rhythmic self-control through MEG-BCI feedback training using a feedback paradigm of limb-motor imagery for binary decision-making communication |
| Chen et al. (2009) [125] | | Subjects followed visual cues to complete the corresponding movements of the left hand, right hand, right foot, and tongue | | | The left and right-hand movements activate the contralateral motor cortex. Leg movements activated the central parietal area. Subjects' tongue movements in showed great variability in activating areas in both hemispheres. Most of these activations were concentrated in the beta band (15-30 Hz) associated with motor intention | Demonstrates that non-invasive MEG-BCI enables reliable multidimensional control of neural prostheses |
| Halme et al. (2016) [126] | | Subjects imagined left or right finger tapping based on visual cues | | | Sensorimotor cortex MEG 10 and 20 Hz oscillations characterize imagined left and right finger tapping | Single-trial MEG decoding MI achieved good accuracy and the online MEG neurofeedback system had good performance |
| Chholak et al. (2019) [127] | | Subjects performed imaginary left or right arm movements based on auditory cues | | | The lower parietal lobe of the kinesthetic imagery (KI) group or the upper parietal and occipital lobe of the visual imagery (VI) group had significant changes in the MEG α (8-12 Hz) and β (15-30 Hz) bands ERD/ERS | It was shown that activation and inhibition of different brain regions (motor-related alpha and beta band power ERD/ERS) could distinguish between KI and VI |
| Rathee et al, (2021) [128] | | Subjects performed four mental imagery tasks (such as hands/feet imagery, subtraction imagery, and word generation imagery) | | | Spatiotime features generated from alpha band (8-12 Hz) and beta band (14-30 Hz) data of MEG signals can characterize MI and cognitive imagery tasks | Published an EEG and EEG-related EEG-based dataset using traditional BCI paradigm recordings involving motor imagery and cognitive imagery tasks |
