# Supplementary material for: Brain-computer interface paradigms and neural coding
Source: Front Neurosci. 2024 Jan 15;17:1345961. doi: 10.3389/fnins.2023.1345961 (PMC10822902; doi:10.3389/fnins.2023.1345961)
Supplement: Supplementary file 6 [file Table_9.DOCX]

Supplementary Material

# Supplementary Tables

Table 6 Examples for existing main EEG-BCI paradigms and neural coding research

| References | Paradigms | Neural Coding | Main Conclusions |
| --- | --- | --- | --- |
| Neuper et al. (1997) [129] | Subjects were asked to imagery left- or right-hand motor imagery to control the moving hand for object-related grasping | Increases of ERDs in the motor cortex were particularly seen in the lower alpha band (8-10 Hz) and beta band (16-20, 20-24 Hz) | Sensorimotor rhythms can be regulated through motor imagery |
| Kaiser et al. (2014) [130] | Subjects were asked to imagine the movement of squeezing a ball with the right hand and tapping with both feet | Motor cortex with stronger event-related desynchronization in higher beta bands | BCI training affects the ability of the cortex to activate, thus potentially promoting and guiding neuroplasticity |
| Ramoser et al. (2000) [131] | Subjects were asked to imagery right- or left-handed movements according to the direction of the arrow | Broadband (for example, 8-30 Hz) changes in the motor cortex after passing through space filters | Spatial filters can effectively extract discriminative information from left- and right-handed motor imagery |
| Pfurtscheller et al. (2003) [132] | Subjects were asked to imagine the movements of the feet | Foot movement imagery produces stable and centrally focused beta oscillations with a primary frequency of 17 Hz | Restoration of hand grasping function in tetraplegic patients by imagining beta oscillations in a task |
| MÜller-Putz et al. (2007) [133] | Subjects were asked to perform passive, active, and imagined foot movements | There is a corresponding decrease and increase in the spectral amplitude of the beta rhythm (13-35 Hz) of the motor cortex before and during exercise | Healthy subjects had significant β-ERD/ERS in the task, but no significant ERD/ERS pattern was found in the paraplegic group |
| Zhang et al. (2023) [134] | Subjects were asked to perform hand movements to the left, center, and right in response to visual cues | Characterization of coordinated movements of both hands based on ERDs associated with motor execution in the alpha band under C3/C4 electrodes | The first decoding of coordinated two-handed movements in the left, center, and right directions was proposed |
| Hashimoto et al. (2013) [135] | Subjects were asked to imagery left and right foot movements | Increase of beta rhythm amplitude in motor cortex at the end of imagery | Unilateral foot movements can be used to discriminate between the left and right foot by increasing the amplitude of the beta rhythm, which can be used to control the neural prosthesis of the foot or the robotic foot. |
| Pfurtscheller et al (1997) [136] | Subjects were asked to imagery movements of the right or left hand based on visual cues | Changes in different frequency components of the alpha band (9-14 Hz) and beta band (18-26 Hz) in motor cortex | Shows significant desynchronization in the contralateral central region and significant synchronization in the ipsilateral side |
| Gernot et al. (2006) [137] | Subjects were asked to place their index finger on the stimulation device and apply different stimulation frequencies (17 to 35 Hz) to the index finger for 2 seconds | When focusing on the stimulation of the right index finger, peaks appeared at 31 Hz at C3 and Cz. When focusing on the left index finger, a peak appeared at 26 Hz at C4 | Using tactile stimuli to stimulate the two index fingers in the "resonance"-like frequency range of the somatosensory system, it is possible to build an SSSEP-BCI |
| Flint et al. (2022) [138] | Subjects were asked to perform rehabilitation experiments by calibrating the force transducers (Factive and Fhaptic) and the forces that can be applied by BMI (FBMI). | Force and voluntary force Factive according to motor cortex γ (70-115 Hz) measured by the Fhaptic for neurorehabilitation BCI control encoded subjects' tactile intentions based on the proportion of both in the total force ε and the MVF scaling factor | Improved synchronization between neuromodulation and force control by high gamma signaling is potentially important for maximizing the ability of nrbmi to induce neural circuit plasticity |
| Furdea et al. (2009) [140] | Subjects were asked to complete spellings based on auditory stimuli assigned to each row and column of verbal numbers | The mean latency of auditory ERP spelling is delayed 150ms-250ms relative to visual ERP spelling | Auditory-evoked ERPs can be reliably categorized |
| Klobassa et al. (2009) [141] | Subjects were asked to use a speller based on six environmental sounds (which were used to represent six columns and six rows) | At the Pz position, the peak amplitude after auditory stimulation was higher than that after non-target stimulation, and the latency was higher than that after non-target stimulation | Auditory P300-BCI may be of great value to people with disabilities who are unable to use a visual BCI |
| Brouwer et al. (2010) [142] | Subjects were asked to follow tactile cues and select tactile triggers designated as targets | A large number of haptics are expected to enhance the amplitude of the P300 in the parietal lobe | Tactile P300-BCI may be suitable for patients with impaired vision or eye movement |
| Cheng et al. (1999) [144] | Subjects were asked to move the cursor according to the instructions of four rectangular blocks flashing at different frequencies around the cursor | When the amplitude threshold of one of the frequency flickers is greater than the threshold of the other frequency flicker on both channels on visual cortex O1 and O2, the corresponding block is considered to have been selected | Helps people with mobility impairments control the movement of a two-dimensional cursor for computer use |
| Chen et al. (2015) [145] | Subjects were asked to spell based on the flicker stimuli | Narrow-band alpha band (8-15.8 Hz) oscillations in (visual cortex) encoded the flicker stimulus pattern | Fundamental and harmonic components significantly improve the performance of SSVEP-BCI, thus facilitating its practical applications such as high-speed spelling |
| Zhang et al.（2021） [146] | Subjects respond based on visual stimuli generated dynamically by machine vision. Narrowband alpha oscillations in the visual cortex encode the flickering stimulus pattern | In the visual cortex, narrowband alpha oscillations (7.2-10Hz) encode the flickering stimulus pattern. | Key parameters in augmented reality (AR) visual stimuli are analyzed, including visual stimulus size, frequency, dynamic object movement speed, and the impact of Information Transfer Rate (ITR) on Brain-Computer Interface (BCI) decoding |
